# Reengineering glycyrrhetinic acid into a therapeutic oligomer for targeted tumor therapy with cardioprotection

**DOI:** 10.1186/s12951-025-03765-5

**Published:** 2025-11-12

**Authors:** Zixin Wang, Bo Su, Alu Ouyang, ZiXuan Liang, Pingyun Yuan, Xin Qin, Yu Li, Xuejing Huang, Ling Fan, Hongwei Guo, Ronghua Jin

**Affiliations:** 1 Guangxi Key Laboratory of Bioactive Molecules Research and Evaluation, Pharmaceutical College, Guangxi Medical University, 22 Shuangyong Road, Nanning, 530021 P R China; 2 Key laboratory of Chemistry and Engineering of Forest Products, Guangxi Key Laboratory of Chemistry and Engineering of Forest Products, Guangxi Collaborative Innovation Center for Chemistry and Engineering of Forest Products, School of chemistry and chemical engineering, State Ethnic Affairs Commission, Guangxi Minzu University, Nanning, 530006 P R China; 3 Guangxi Medical University Cancer Hospital, Guangxi Medical University, No. 71 Hedi Road, Nanning, 530021 P R China; 4 Shaanxi Key Laboratory of Biomedical Metal Materials, Northwest Institute for Nonferrous Metal Research, Xi’an, 710016 P. R. China

**Keywords:** Poly-glycyrrhetinic acid, Polymeric nanodrug, Glycyrrhetinic acid, Hepatocellular carcinoma, Cardiotoxicity

## Abstract

To overcome the inherent limitations of conventional nanocarriers such as therapeutic inefficacy and suboptimal drug loading capacity, a novel “polymeric nanodrug” strategy was developed. This approach redefines nanodrug design by employing glycyrrhetinic acid (GA) simultaneously as a bioactive agent and a polymerizable targeting monomer to synthesize poly-glycyrrhetinic acid (PGA), achieving near-theoretical drug loading approaching 100%. Subsequent PEGylation yielded hepatocellular carcinoma (HCC)-targeting PGA-PEG-GA nanodrugs, which exhibited a 1.89-fold enhancement in antitumor efficacy compared to free GA. The exceptionally low critical micelle concentration (CMC) of PGA-PEG-GA enables efficient encapsulation of additional therapeutics. Using doxorubicin (DOX) as a model drug, HCC-targeting nanodrugs (denoted as DOX@PGA-PEG-GA NDs) were fabricated. In vitro studies demonstrated that the nanodrugs induced 2.5-fold higher toxicity in tumor cells than normal cells, enhanced cellular uptake by 3.0-fold, and reduced DOX-induced cardiomyocyte apoptosis by 54%. In vivo evaluations revealed a tumor inhibition rate of 89.7 ± 5.2% for DOX@PGA-PEG-GA NDs and 40.09 ± 11.94% for blank PGA-PEG-GA NDs, together with 18.3-fold higher intra-tumoral accumulation compared to free DOX and complete mitigation of DOX-related cardiotoxicity. This integrated “structure-function-safety” strategy effectively overcomes critical challenges in drug-loading efficiency and functional synergy, offering a robust and novel platform for targeted nanodrug applications.

**Graphical Abstract:**

**Figure:**
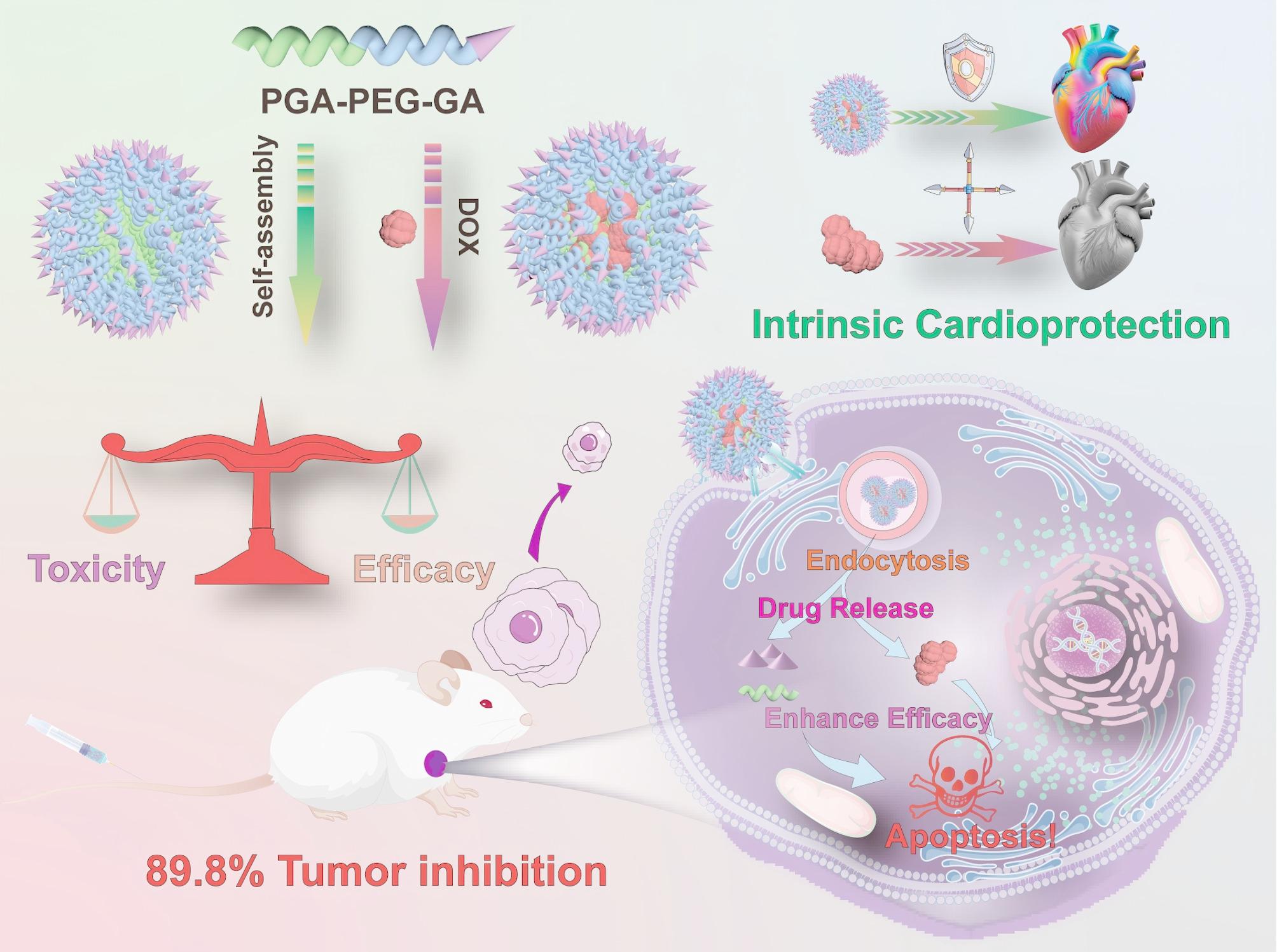
Schematic illustration of the fabrication of DOX@PGA-PEG-GA NDs and its role in enhancing the anti-tumor efficacy and cardioprotective function. The therapeutic mechanism of the micellar system involves GA-mediated cellular endocytosis, where subsequent esterase-triggered degradation under acidic pH conditions enables synchronized release of DOX and GA. This dual-drug delivery system demonstrates synergistic antitumor efficacy through complementary pharmacological actions, while the co-released GA effectively attenuates DOX-induced cardiotoxicity by modulating oxidative stress pathways. The integrated process thereby achieves concurrent tumor suppression and cardioprotective intervention through a single administration protocol.

**Supplementary Information:**

The online version contains supplementary material available at 10.1186/s12951-025-03765-5.

## Introduction

The U.S. Food and Drug Administration has approved 62 nanodrugs for clinical use, with more than 500 additional candidates currently under active clinical investigation, 53% of which target malignant tumors [1, 2]. Despite these advances, conventional nanocarriers face two fundamental scientific challenges: (1) low drug loading capacity with average loading efficiency typically below 10% (w/w)) [3]. Achieving therapeutic dosages often requires excessive excipient administration, which may contribute to systemic metabolic burden. (2) Functional disjunction between nanocarriers and drugs, a limitation inherent in many passively loaded nanocarriers [4–7]. These nanocarriers not only lack intrinsic antitumor activity but also produce toxic degradation by-products. Even clinically established formulations such as DOXIL^®^ exhibit limited drug loading (11% w/w), while their conventional lipid excipients present long-term safety concerns [8]. To overcome these limitations, the development polymeric nanodrugs with integrated drug-carrier functionality and intrinsically bioactive materials has become a crucial research direction for enabling next-generation precision nanodrugs.

The rational design of polymeric nanodrugs based on highly efficient, low-toxicity therapeutic molecules with intrinsic pharmacological activity offers considerable scientific and translational promise [9]. Natural compounds, in particular, have attracted extensive research interest due to their remarkable therapeutic use in treating human diseases for thousands of years [10–12]. Glycyrrhetinic acid (GA), the primary bioactive component of licorice, exhibits multifaceted antitumor efficacy through diverse mechanisms such as apoptosis induction, cell cycle arrest, metastasis suppression, immunomodulation, and chemosensitization [13, 14]. Mechanistically, GA act as a substrate for protein kinase C (PKC) [15] and specifically binds to PKC, which is overexpressed in hepatocellular carcinoma (HCC), facilitating tumor-selective accumulation [16]. Clinically, six topical GA-based formulations have been approved for dermatological use, with two additional GA formulations currently in phase I/II trials (JPRN-jRCTs031220306, NCT05570110), further supporting its safety profile and therapeutic potential [17]. Capitalizing on the “drug-as-carrier” concept [18, 19], we incorporated GA as functional monomers into polymer backbones via enzyme-responsive ester bonds, achieving exceptional drug loading capacity and improved therapeutic efficacy. This molecular integration bridges natural compounds with nanotechnology, positioning GA-derived polymers as a transformative platform for precision oncology.

To further optimize the performance of PGA, an HCC-targeting amphiphilic block copolymer, PGA-PEG-GA, was engineered. While previous studies have employed physiologically active molecules to construct polymeric systems, such designs often require additional stabilizers such as DSPE-PEG to form the nanoparticles and generally lack intrinsic active targeting capabilities [20–22]. Moreover, high-molecular-weight polymers in these systems often hinder efficient hydrolytic release of the active monomers. Although PGA-PEG-GA exhibits a relatively low degree of polymerization (DP), its molecular weight exceeds that of Genexol^®^-PM, which is already clinically available. More importantly, PGA-PEG-GA displays a low critical micelle concentration (CMC = 0.0076 g/L), lower than those of several FDA-approved surfactants [23–28], indicating superior stability and considerable potential for drug encapsulation. The relatively low DP of PGA-PEG-GA also facilitates efficient hydrolytic release of the bioactive GA. Doxorubicin (DOX), a broad-spectrum chemotherapeutic agent, demonstrates significant clinical efficacy but suffers from dose-dependent cardiotoxicity that severely limits its utility [29–32]. Notably, co-administration of free GA with DOX has been reported to synergistically enhance antitumor efficacy while alleviating myocardial injury [33, 34]. However, whether PGA-PEG-GA can serve as an effective nanocarrier for DOX to reduce its cardiotoxicity remains unexplored. The triple therapeutic functionality of PGA-PEG-GA as a DOX nanocarrier, an intrinsic antitumor agent, and a potential cardioprotectant warrants further mechanistical investigation to fully elucidate its multifaceted pharmacological interactions.

**Fig. 1:**
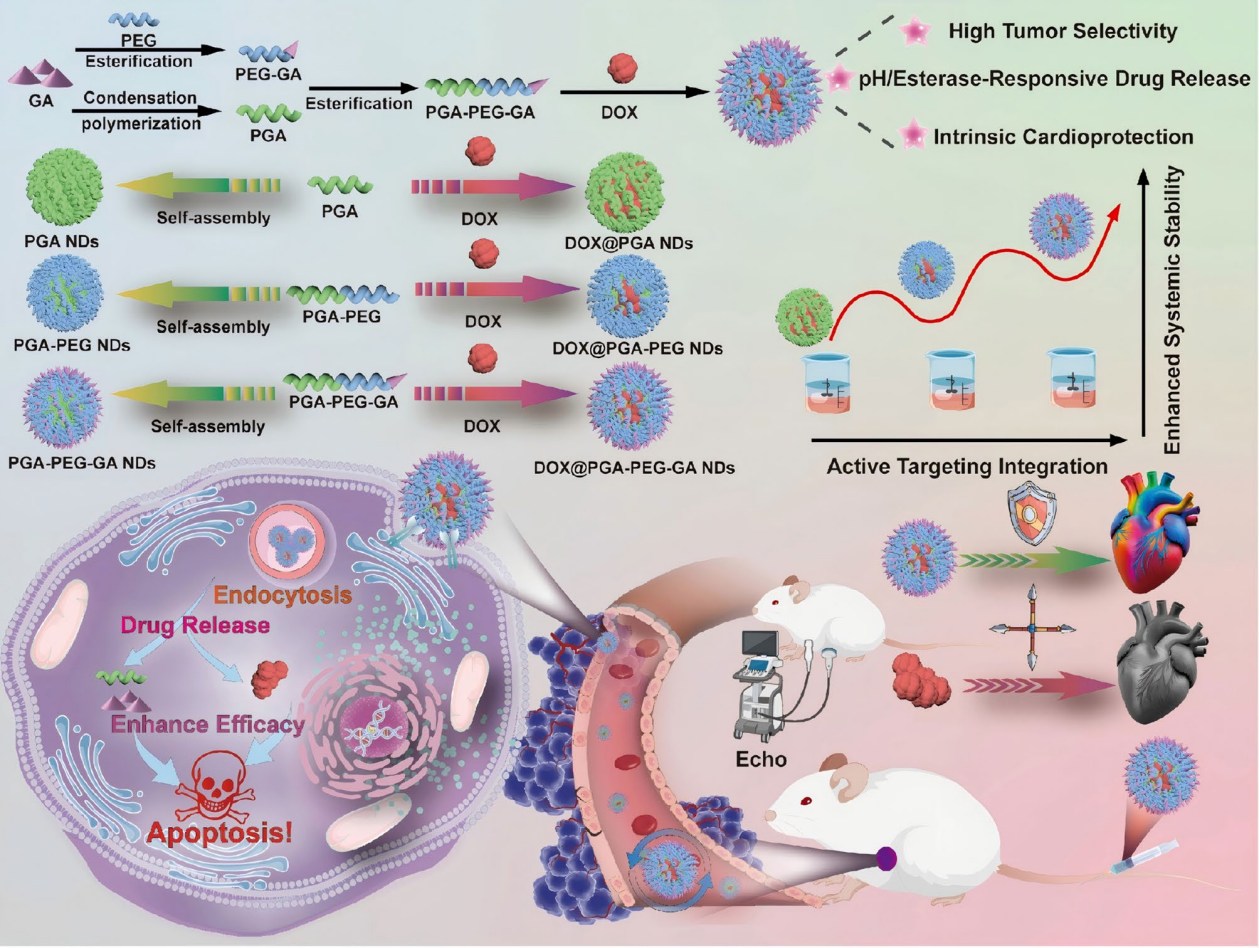
Schematic illustration of the synthesis of DOX@PGA-PEG-GA NDs and its role in enhancing the anti-tumor efficacy and cardioprotective function

In this study, we developed an HCC-targeting amphiphilic block copolymer, PGA-PEG-GA, using GA as a polymerizable therapeutic monomer that confers both intrinsic antitumor activity and targeting capability towards HCC. The resulting PGA-PEG-GA exhibited potent antitumor efficacy, achieving a 1.89-fold increase in anti-tumor activity compared to free GA. Mechanistic studies into its self-assembly behavior were conducted using molecular dynamics simulations and assembly-disrupting agents. DOX-loaded PGA-PEG-GA nanodrugs (DOX@PGA-PEG-GA NDs) demonstrated 3.0-fold greater cellular internalization in HepG2 cells compared to normal hepatocytes, 18.3-fold higher tumor accumulation than free drug molecules at 24 h, and reduced cardiac biomarkers to normal levels in both in vitro and in vivo models. This work establishes a “drug-as-carrier” nanotherapeutic platform that synergistically combines tumor-selective targeting, intrinsic bioactivity, and systemic toxicity reduction (Fig. 1).

## Experimental section

### Synthesis of Poly (Glycyrrhetinic Acid) (PGA)

Poly (glycyrrhetinic acid) (PGA) was synthesized via low-temperature polycondensation using GA as the monomer, thionyl chloride (SOCl₂) as the acylating agent, pyridine as the acid scavenger, and anhydrous N, N-dimethylformamide (DMF) as the solvent. In a typical procedure, GA (1.0 g) was dissolved in a mixture of anhydrous DMF (5 mL) and pyridine (5 mL) under vigorous stirring in an ice bath (0–5 °C). SOCl₂ was added dropwise to initiate esterification. Key reaction parameters including polymerization duration (0.5–4 h) and the GA-to-SOCl₂ molar ratios (2:1, 1:1, 1:2, 1:4) were systematically optimized to investigate their effects on molecular weight (Mw) and polydispersity (PDI). After quenching the reaction with deionized water (20 mL), the mixture was extracted with dichloromethane (3 × 20 mL). The combined organic layers were dried over anhydrous sodium sulfate, filtered, and concentrated under reduced pressure using a rotary evaporator. The crude product was purified through three cycles of dispersion in cold methanol (10 mL per cycle) followed by centrifugation (10,000 rpm, 15 min) to remove unreacted precursors. Finally, the purified PGA was obtained as a white powder via lyophilization.

## Synthesis of glycyrrhetinic acid functionalized PEG (PEG-GA)

PEG-GA was synthesized in a dried 25 mL round-bottom flask under a nitrogen atmosphere. GA (0.50 g) was dissolved in anhydrous tetrahydrofuran (THF, 10 mL) and cooled to 0–5 °C in an ice bath. Thionyl chloride (SOCl₂, 150 µL) was added dropwise, and the mixture was stirred at 0–5 °C for 1 h to activate carboxylic acid groups, followed by further stirring at room temperature for 23 h to complete acylation. The reaction mixture was concentrated by rotary evaporation to remove excess SOCl₂ and THF, yielding activated GA as a crude intermediate. For conjugation with PEG (2.34 g, Mn = 2000), the PEG was dissolved in a mixture of anhydrous dichloromethane (DCM, 5 mL) and pyridine (5 mL) under ice cooling. The activated GA in DCM (5 mL) was added dropwise to the PEG solution over 30 min, and the resulting mixture was stirred at 0–5 °C for 1 h and then at room temperature for 23 h. The crude product was concentrated under reduced pressure and purified by silica gel column chromatography with gradient elution (DCM/MeOH, 100:1→10:1, v/v). PEG-GA was obtained as a pale-yellow viscous product (2.28 g, 78.3% yield), and its structural was confirmed by ¹H-NMR, FT-IR, and UV-Vis spectroscopy.

## Synthesis of Poly (Glycyrrhetinic acid)-PEG-Glycyrrhetinic acid (PGA-PEG-GA)

PGA-PEG-GA was synthesized under optimized PGA polymerization conditions. Following the polycondensation of GA using an optimized GA-to-SOCl₂ molar ratio for 0.5 h, PEG-GA (0.9 g) was added to the reaction mixture under a nitrogen atmosphere. The mixture was stirred in an ice bath (0–5 °C) for 1 h, and then at 25 °C for 23 h to facilitate copolymerization. The reaction was quenched with 20 mL of deionized water and extracted with dichloromethane (3 × 20 mL). The combined organic layers were dried over anhydrous sodium sulfate, filtered, and concentrated under reduced pressure using a rotary evaporation. The crude product was purified by dropwise precipitation into ice-cold diethyl ether (200 mL), followed by three cycles of dispersion and centrifugation (10,000 rpm, 15 min, 4 °C). Further purification was performed by dialysis (MWCO: 3.5 kDa) against methanol for 24 h and water for 48 h, with the dialysate replaced every 6 h. The solution was frozen at − 80 °C and lyophilized for 72 h to obtain PGA-PEG-GA as a pale-yellow solid (1.03 g, yield: 54.2%). For the synthesis of PGA-PEG, an identical procedure was employed, except that PEG-GA was substituted with methoxy polyethylene glycol (m-PEG, 0.7 g), yielding a grayish-white solid (0.99 g, yield: 58.5%). The final products were characterized by ¹H-NMR, FT-IR, and gel permeation chromatography (GPC) to confirm copolymer structure, purity, and molecular weight distribution.

## X-ray diffraction (XRD)

Appropriate amounts of GA, PGA, PEG, and PGA-PEG-GA were compressed into thin discs. X-ray diffraction analysis was then performed with a scan range of 5° to 80°.

## Critical micelle concentration (CMC)

The CMC of PGA-PEG-GA was determined using pyrene fluorescence spectroscopy. Briefly, 0.1 g of PGA-PEG-GA was dissolved in 3 mL of tetrahydrofuran (THF) and added dropwise to 50 mL of deionized water under continuous stirring. The THF was removed by rotary evaporation at 25 ℃ for 3 h, and the resulting residue was diluted to 100 mL to obtain a micelle stock solution with a concentration of 1.0 g/L. Simultaneously, 12 mg of pyrene was dissolved in acetone, after which the solvent was evaporated. A 100 µL aliquot of this solution was transferred to a volumetric flask and dried at 50 °C. The micelle solution was serially diluted to concentrations ranging from 0.5 to 0.0001 g/L and added to the pyrene-containing flasks to achieve a final pyrene concentration of 6 × 10⁻⁷ mol/L. The samples were equilibrated at 50 °C for 12 h followed by 2 h at 25 °C. Fluorescence spectra were acquired by scanning excitation wavelengths from 290 to 350 nm with emission fixed at 373 nm. The micelle concentration was determined from the plot of the intensity ratio I_336_/I_334_ versus micelle concentration by identifying the intersection point of the fitted curve.

### Synthesis and characterization of RhB or DOX@PGA-PEG-GA NDs

DOX@PGA-PEG-GA NDs were prepared using a nanoprecipitation method. Briefly, Solution A was formed by dissolving PGA-PEG-GA (20.0 mg) in DMSO (1.0 mL) via ultrasonication. Solution B was prepared by dissolving DOX·HCl (2.0 mg) in DMSO, followed by the addition of an equimolar amount of triethylamine and ultrasonication for 30 min. Solutions A and B were then combined under continuous stirring and injected dropwise into deionized water under vigorous agitation to form a nanoparticle suspension. The suspension was dialyzed at room temperature to remove residual solvents and subsequently lyophilized to obtain DOX@PGA-PEG-GA NDs. For control samples, DOX@PGA-PEG NDs and DOX@PGA NDs were synthesized under the same procedure using PGA-PEG or PGA as the polymeric matrix, respectively. Blank nanodrugs were prepared following the identical process without the addition of DOX.

Twenty milligrams of PGA-PEG-GA and 2 mg of rhodamine B (RhB) were dissolved in 1 mL of dimethyl sulfoxide (DMSO). The solution was then added dropwise to deionized water under vigorous stirring to induce nanoprecipitation. The resulting suspension was dialyzed at room temperature to eliminate free RhB and residual DMSO, and subsequently lyophilized to obtain purified RhB@PGA-PEG-GA NDs.

The hydrodynamic diameter and polydispersity index (PDI) of the nanodrugs were determined by dynamic light scattering (DLS) at 25 °C. Morphological characterization was carried out using scanning electron microscopy (SEM) following platinum sputtering. Colloidal stability was evaluated by dispersing nanodrugs (0.1 mg/mL) in phosphate-buffered saline (PBS, pH 7.4), DMEM cell culture medium, or DMEM supplemented with 10% fetal bovine serum (FBS). The hydrodynamic diameter and zeta potential were monitored via DLS at predetermined time intervals.

## Determination of encapsulation efficiency and Drug-Loading capacity

The encapsulation efficiency (EE) and drug-loading capacity (DLC) of the nanodrugs were determined using a fluorescence spectrophotometer. A sample of 0.1 mg of drug-loaded nanodrugs was completely dissolved in 10 mL of dimethyl sulfoxide (DMSO). Then, 1 mL of this solution was transferred to a quartz cuvette for fluorescence measurement. The drug content was quantified based on fluorescence emission at 600 nm after excitation at 480 nm. The encapsulation efficiency and drug-loading capacity were calculated using the following formulas:

1 $$\begin{aligned}&\ \text{EE} \ (\text{Weight} {\%}) \\&\quad= \text{(mass of encapsulated drug / mass of added drug)} \\&\quad \times100 {\%}\end{aligned}$$

2 $$\begin{aligned}\text{DLC} & \,(\text{weight} {\%}) \\&\quad= \text{(mass of encapsulated drug / total mass of NDs)} \\&\quad\times100{\%}\end{aligned}$$

## In vitro drug release profiles

The release kinetics of the encapsulated drug were evaluated using a dialysis-based method. Briefly, 1 mL of DOX@PGA-PEG-GA NDs was placed in a dialysis bag (MWCO: 3.5 kDa) with or without porcine liver esterase (50 U/mL) [35]. The bags were immersed in 20 mL of buffer solutions at different pH values (4.0, 6.8, and 7.4) and incubated at 37 °C under gentle stirring at 100 rpm. The concentration of DOX was quantified by fluorescence measurement at excitation and emission wavelengths of 480 nm and 600 nm, respectively. The concentration of GA was determined via UV absorption spectroscopy.

### Hemolysis

Red blood cells (RBCs) isolated from Kunming mice were diluted to a 2% (v/v) suspension in phosphate-buffered saline (PBS). DOX@PGA-PEG-GA NDs were then introduced into the RBC suspensions. The mixtures were incubated at 37 °C for 3 h and subsequently centrifuged at 1500 rpm for 15 min to sediment the cells. The resulting supernatant was analyzed by UV spectrophotometry between 450 and 700 nm. The hemolysis rate was calculated using the standard formula.

3 $$\begin{aligned}&\text{Hemolysis rate}\,({\%}) \\&\quad = (\text{A}_\text{sample} - \text{A}_{0}) / (\text{A}_{100}-\text{A}_{0})\\&\quad \times 100 {\%}\end{aligned}$$

Here, A_sample_ represents the absorbance of the test sample, A_100_ refers to the absorbance of the positive control (fully lysed RBCs in water), and A_0_ corresponds to the absorbance of the negative control (PBS with 0% hemolysis), collectively enabling the quantitative assessment of hemolytic activity.

### Molecular dynamics (MD) simulation

The simulation system was constructed with the PACKMOL package [36] by placing 200 of PGA-PEG-GA, 7 molecules of DOX, 100 Na^+^ ions, and 100 Cl^−^ ions within the simulation box. The OPLS-AA force field was applied to model all components [37, 38]. Periodic boundary conditions were employed with a cutoff radius was 1.2 nm. Long-range electrostatic interactions were treated using the Ewald method. Prior to MD simulation, the system underwent annealing by cycling the temperatures from 300 to 500 K in the NPT (*P* = 101 KPa) ensemble for 500 ps to achieve equilibration. All initial configurations were energy-minimized using the steepest descent algorithm. Temperature and pressure were regulated with the Nose-Hoover thermostat and Parrinello-Rahman barostat, respectively. Finally, MD simulation was conducted in the NVT ensemble (T = 300 K) for 1000 ps to obtain the production trajectories.

Nonbonded interactions include van deer Waals (vdW) and electrostatic terms, which are described by Eqs. 4 and 5, respectively.

4 $$\text{E}_{LJ}(r_{ij}) = 4\epsilon_{ij}\left(\left(\frac{\sigma_{ij}}{r_{ij}}\right)^{12} - \left(\frac{\sigma_{ij}}{r_{ij}}\right)^{6}\right)$$

5 $$E_r\!\left(r_{ij}\right)=\frac{q_i q_j}{4\pi\varepsilon_0\varepsilon_r\,r_{ij}}$$

In the equation, $\:{q}_{i}$、$\:{q}_{j}$ represent atomic charges, $\:{r}_{ij}\:$ denotes the interatomic distance, $\sigma$ is the atomic diameter, and $\:\epsilon\:$ represents the energy well-depth parameter.

For different atom types, the van deer Waals (vdW) interactions were described using geometric mixing rules, as defined in Eq. 6. A cutoff distance of 1.2 nm was applied to both vdW and electronic interactions, and the Particle–Particle Particle–Mesh (PPPM) method was used to calculate long-range electrostatic contributions.

6 $$\:{\sigma\:}_{ij}=\frac{1}{2}\left({\sigma\:}_{ii}+{\sigma\:}_{jj}\right);{\epsilon\:}_{ij}={\left({\epsilon\:}_{ii}\text{*}{\epsilon\:}_{jj}\right)}^{\frac{1}{2}}$$

#### Self-Assembly mechanism

The self-assembly behavior of the nanodrugs was investigated using selective inhibitors targeting distinct intermolecular forces: sodium chloride (NaCl) for electrostatic interactions, Triton X-100 for hydrophobic interactions, and urea for hydrogen bonding. A stock solution of the nanodrugs was prepared at a concentration of 0.1 mg/mL. Then, 0.1 mL aliquots were transferred into 1.5 mL centrifuge tubes and mixed with varying concentrations (0, 1, 5, 10, 50, 100 mM) of NaCl, Triton X-100, or urea, adjusting the final volume to 1 mL per tube. After incubation at ambient temperature for 2 h, changes in nanoparticle size were measured using a nanoparticle size analyzer to elucidate the driving forces underlying the self-assembly process.

### Cellular uptake

Human cardiac myocytes (HCM) were purchased from ScienCell Research Laboratories (Shanghai, China). The cells were cultured in high-glucose DMEM supplemented with 10% fetal bovine serum (FBS), penicillin (10 kU/mL), streptomycin (10 mg/mL), and amphotericin B (25 µg/mL). All cultures were maintained at 37 °C in a humidified atmosphere containing 5% CO₂.

Cellular uptake of DOX@PGA-PEG-GA NDs was evaluated in HepG2 and LX-2 cells via flow cytometry. Cells were seeded in 6-well plates at a density of 2 × 10⁵ cells per well and allowed to adhere overnight. The culture medium was then replaced with fresh medium containing DOX@PGA-PEG-GA NDs, and the cells were incubated for 1, 2, or 4 h at 37 °C under 5% CO₂. Untreated cells were used as negative controls. After incubation, the cells were washed twice with ice-cold PBS to remove non-internalized nanodrugs and detached using 0.25% trypsin-EDTA. The resulting cell suspensions were centrifuged at 1,000 rpm for 5 min and resuspended in PBS for analysis on a BD Accuri C6 Plus flow cytometer. DOX fluorescence was detected in the PL2-A channel (Ex/Em: 488/575 nm), with 1 × 10^4^ cells recorded per sample. Data was analyzed using FlowJo software (v10.8.1) to quantify the percentage of DOX-positive cells.

### In vitro lysosomal colocalization

The intrinsic fluorescence of DOX encapsulated in PGA-PEG-GA NDs was utilized to assess its colocalization with lysosomes within HepG2 cells using confocal laser scanning microscopy (CLSM; FV3000, Olympus). Cells were seeded at a density of 1 × 10^5^ cells per well in confocal dishes (NEST) and cultured for 24 h. Subsequently, the cells were treated with either free DOX or DOX@PGA-PEG-GA NDs dispersed in DMEM for 1, 2, and 4 h. After treatment, the medium was aspirated, and the cells were washed three times with PBS. Lysosomes were stained by incubation with LysoTracker Red (100 nM) for 30 min, and nuclei were stained with Hoechst 33342 (10 µg/mL) for 10 min. Following two additional washes with PBS (pH 7.4), the cells were imaged by CLSM. The blue, green, and red channels were used to visualize nuclei, DOX fluorescence, and lysosomes, respectively, allowing detailed analysis of the intracellular distribution of the nanodrugs.

For quantitative assessment of cellular uptake, flow cytometry was performed using a BD Accuri C6 Plus instrument. HepG2 cells were plated in 6-well plates at 2 × 10^5^ cells per well and cultured for 24 h. The cells were then treated with free DOX or DOX@PGA-PEG-GA NDs for 1, 2, or 4 h, with untreated cells serving as the negative control. After incubation, the cells were rinsed twice with cold PBS (pH 7.4) to remove debris, and cellular uptake was quantified by measuring the percentage of DOX-positive cells via flow cytometry.

### In vitro cytotoxicity assay

The CCK8 assay was employed to evaluate the cytotoxicity of free GA, free DOX, PGA NDs, DOX@PGA NDs, PGA-PEG NDs, DOX@PGA-PEG NDs, PGA-PEG-GA NDs, and DOX@PGA-PEG-GA NDs in HepG2 (human hepatocellular carcinoma), H22 (mouse hepatocellular carcinoma), and LX-2 (human normal liver) cells. After trypsinization and counting, cells were seeded into 96-well plates at a density of 5000 cells per well and incubated for 24 h at 37 °C in a 5% CO_2_. The cells were then treated with each formulation at various concentrations: blank nanodrugs were tested at 10, 20, 40, 60, and 80 µg/mL, while DOX loaded nanodrugs were dosed base on DOX equivalence (0.5, 1, 1.5, 2, and 4 µg/mL), with 200 µL per well. Following 48 h treatment, 10 µL of CCK8 reagent was added to each well, and the plates were incubated for an additional 4 h. Absorbance was measured at 450 nm using a microplate reader. Cell viability was calculated as a percentage relative to untreated controls by comparing the optical densities.

The data was analyzed by nonlinear regression to get the IC_50_ value. The combination index (CI) values were calculated by the equation [39]: CI = C_A, comb_/C_A, alone_ + C_B, comb_/C_B, alone_. C_A, alone_ and C_B, alone_ represent the concentrations of each drug required to achieve the IC₅₀ when administered alone, while C_A, comb_ and C_B, comb_ denote the actual concentrations of the respective drugs in the combination treatment at which the IC₅₀ is reached. Using this analysis method, a CI = 0.9 − 1.1 reflects additive activity, and a CI >1.1 indicates antagonism, while a CI < 0.9 suggests synergy.

### Cell apoptosis assay

Cells were seeded in 6-well plates at a density of 1 × 10^5^ cells per well in 2 mL of DMEM supplemented with 10% FBS, and allowed to adhere for 48 h. The cells were then treated for 24 h with DMEM containing free GA, free DOX, PGA NDs, DOX@PGA NDs, PGA-PEG NDs, DOX@PGA-PEG NDs, PGA-PEG-GA NDs, or DOX@PGA-PEG-GA NDs. Blank nanodrugs were applied at a concentration of 20 µg/mL, while DOX-equivalent nanodrugs were used at 1.5 µg/mL. After treatment, the cells were detached using trypsin solution without ethylenediaminetetraacetic acid (EDTA). According to the manufacturer’s protocol (Beyotime, Shanghai, China), cells were collected and resuspended in 400 µL of assay buffer, incubated with 5 µL of Annexin V-FITC for 15 min in the dark, and then stained with 5 µL of propidium iodide (PI) for 5 min, also protected from light. Finally, 2 × 10^4^ cells per sample were acquired and analyzed by flow cytometry to quantify apoptosis.

### Live/dead cell staining

Live/dead cell staining was carried out using calcein acetoxymethyl ester (Calcein-AM) and propidium iodide (PI). HepG2 cells were seeded in 6-well plates and cultured overnight to allow adherence, followed by treatment according to the groups established in the CCK-8 assay. After 48 h of treatment, the cells were detached using 0.25% trypsin-EDTA, washed twice with PBS, and resuspended in a staining solution containing Calcein-AM (2 µM) and PI (1.5 µM). The cell suspension was incubated in the dark at 25 °C for 30 min, then transferred to confocal dishes for imaging. Viable cells were identified by green fluorescence from Calcein (excitation/emission: 494/517 nm), whereas dead cells were indicated by red fluorescence from PI (excitation/emission: 535/617 nm). Images were acquired using a laser scanning confocal microscope under standardized parameters.

### Lactate dehydrogenase (LDH)

An LDH standard curve was prepared by diluting 12 µL of LDH stock solution (2.5 U/mL) with 588 µL of LDH assay buffer to obtain a 50 mU/mL standard solution. Serial dilutions (0, 5, 10, 20, 40, 60, 80, 100 µL) of this standard were then added to a 96-well plate, and each well was adjusted to a final volume of 100 µL using LDH assay buffer, resulting in LDH concentrations of 0, 0.5, 1, 2.5, 5, 10, and 20 mU/mL (equivalent to 0, 0.05, 0.1, 0.25, 0.5, 1, and 2 mU per well). Human cardiomyocytes (HCM) were seeded in 6-well plates and cultured until 80–90% confluency was reached. The medium was then aspirated, and the cells were gently washed with PBS. Four experimental groups were included: (1) control (cell-free medium), (2) untreated cells (sample control), (3) untreated cells incubated with LDH release reagent (maximum enzyme activity control), and (4) drug-treated cells (GA, DOX, GA + DOX, DOX@PGA-PEG-GA NDs, using doses consistent with CCK-8 assays). For the maximum activity control, 10% (v/v) LDH release reagent was added to the wells 1 h prior to measurement. The plates were gently agitated and returned to the incubator. At the indicated time point, 120 µL of supernatant from each well was transferred to a new 96-well plate. LDH activity was measured spectrophotometrically at 490 nm in accordance with the manufacturer’s instructions.

### Reactive oxygen species (ROS)

Cells were seeded in confocal dishes at an optimal density and allowed to adhere overnight. Upon reaching 70–80% confluency, the cultures were treated with GA, DOX, GA + DOX, or DOX@PGA-PEG-GA NDs to induce ROS generation. The ROS-sensitive probe DCFH-DA was diluted to 10 µM in serum-free medium, applied to the cells, and incubated at 37 °C in the dark for 30 min to facilitate intracellular esterase-mediated conversion to DCFH. Excess probe was removed by washing three times with serum-free medium. Nuclei were counterstained with Hoechst 33342 (10 µg/mL in PBS) for 10 min at 25 °C, followed by two washes with PBS. Cellular fluorescence was visualized using a laser scanning confocal microscope, with DCF (ROS indicator) detected at Ex/Em 488/525 nm and Hoechst 33342 at 405/461 nm.

### Mitochondrial membrane potential assay

Cells were plated in confocal dishes and cultured until they reached 60–70% confluence. Following this, the cells were treated with GA, DOX, GA + DOX, or DOX@PGA-PEG-GA NDs according to experimental groups. The JC-1 staining working solution was prepared according to the manufacturer’s instructions: 50 µL JC-1 (200×) was diluted in 8 mL PBS, and then 2 mL of JC-1 staining buffer (5×) was added with thorough mixing. After removal of the culture medium, the cells were gently rinsed 2–3 times with PBS. Then, 1 mL of the JC-1 working solution was added to each dish and incubated at 37 °C for 20 min in a cell culture incubator. After incubation, the supernatant was carefully aspirated, and the cells were washed three times with JC-1 staining buffer (1×). Finally, 1 mL of PBS was added to each dish, and changes in mitochondrial membrane potential were assessed by laser scanning confocal microscopy.

#### Animals

Kunming mice (SPF grade, 4–6 weeks old) were obtained from the Experimental Animal Center of Guangxi Medical University (Guangxi, China). All animal procedures were conducted in compliance with the U.S. National Institutes of Health Guide for the Care and Use of Laboratory Animals and were approved by the Institutional Animal Care and Ethics Committee of Guangxi Medical University (Ethics Approval No: 202310021).

### In vivo biodistribution of PGA-PEG-GA NDs

The biodistribution of the nanodrugs was evaluated using Rhodamine B(RhB) as a fluorescent probe. Briefly, Kunming mice (6 weeks old, ≈ 30 g) were subcutaneously inoculated with 1 × 10^6^ H22 cells. Once the tumor volume reached approximately 200 mm^3^, the tumor-bearing mice were randomly divided into two groups (*n* = 3) and administered either RhB-loaded PGA-PEG-GA NDs or free RhB via tail vein injection at a dose of 0.25 mg/kg RhB in a volume of 200 µL. Under general anesthetic, the mice were imaged at predetermined time points (0, 0.5, 1, 3, 6, 9, 12, 24, and 48 h) using an in vivo imaging system (Aniview 600; Guangzhou Biolight Biotechnology Co., Ltd) with an excitation wavelength at 535 nm and an emission wavelength of 600 nm. At the end of the experiment, the mice were euthanized humanely, and major organs (heart, liver, spleen, lung, and kidney) as well as tumors were collected for ex vivo fluorescence imaging.

#### In vivo antitumor activity of DOX@PGA-PEG-GA NDs

To evaluate the therapeutic efficacy of DOX@PGA-PEG-GA NDs in vivo, H22 cells (1 × 10^6^) were subcutaneously inoculated into the right lateral thoracic region of mice. When the average tumor volume reached approximately 50–100 mm^3^, the mice were randomly allocated into the following treatment groups (*n* = 5 per group): saline, PGA-PEG-GA NDs, free DOX, DOX@PGA NDs, DOX@PGA-PEG NDs, and DOX@PGA-PEG-GA NDs. The mice received intravenous injections of each treatment every three days for a total of five administrations at a DOX-equivalent dose of 5 mg/kg. The saline-treated group served as the negative control. Tumor volumes were measured every two days using calipers, and calculated according to the formula: V = (Length×Width^2^)/2. Meanwhile, body weight was monitored throughout the study to assess systemic toxicity.

#### Pharmacokinetics assay

Kunming mice (≈ 30 g) were randomly divided into two treatment groups: free DOX and DOX@PGA-PEG-GA NDs. Each group included three mice per time point. All animals received a single intravenous injection via the tail vein at a DOX-equivalent dose of 5 mg/kg. At predetermined time points post-injection, three mice from each group were euthanized for blood collection. Blood (~ 1 mL) was obtained via retro-orbital puncture and treated with heparin. The samples were centrifuged at 8000 rpm for 10 min at 4 °C to isolate plasma. The DOX concentration in the plasma was quantified by fluorescence measurement using a multimode microplate reader (Ex: 480 nm, Em: 600 nm).

#### Echocardiography (ECHO)

On day 20 post-treatment, echocardiography was performed using a Canon Aplio i800 system. Mice were anesthetized with isoflurane and maintained under anesthesia using a ventilator, with the total anesthesia time not exceeding 30 min. Cardiac contractile function was assessed using a color Doppler ultrasound system. For left ventricular functional assessment in the long-axis view, ultrasound gel was applied to the probe. An M-mode image of the cardiac long-axis was acquired at the 11 o’clock position. Left ventricular end-systolic diameter (LVESD) and left ventricular end-diastolic diameter (LVEDD) were measured from the images. End-diastolic volume (EDV) and end-systolic volume (ESV) were then calculated based on standard formulas, from which left ventricular fractional shortening (LVFS) and left ventricular ejection fraction (LVEF) were derived. The equations used were as follows:

7 $$\:\text{EDV=}\frac{\text{7.0}}{\text{2.4+LVEDD}}\times\text{LVEDD}\text{3}$$

8 $$\:\text{ESV=}\frac{\text{7.0}}{\text{2.4+LVESD}}\times\text{LVESD}\text{3}$$

9 $$\:\text{LVEF}(\%)=\frac{\text{EDV-ESV}}{\text{EDV}}\times{100}{\%}$$

10 $$\:\text{LVFS}(\%)=\frac{\text{LVESD-LVEDD}}{\text{LVESD}}\times{100}{\%}$$

### Histological examination

Major organs were collected and fixed in 4% paraformaldehyde (PFA) at 4 °C for 24 h. The tissues were then paraffin-embedded, sectioned at 5 μm thickness, and stained with hematoxylin and eosin (HE) for histopathological evaluation [40]. Tumor and heart tissues were snap-frozen in optimal cutting temperature (OCT) compound over dry ice, cryosectioned at 10 μm, and mounted onto adhesive slides. DOX accumulation was quantified by detecting red fluorescence signals (Ex/Em: 480/600 nm) using a fluorescence microscope. For reactive oxygen species (ROS) detection, heart sections were incubated with 1 µmol/L dihydroethidium (DHE) in a humidified chamber at 37 °C for 30 min, washed twice with PBS, and coverslipped with antifade mounting medium. Apoptosis was assessed in tumor and cardiac tissues using a TUNEL Apoptosis Detection Kit (Servicebio, China) according to the manufacturer’s protocol.

### Blood chemistry

Blood samples were collected from the retro-orbital plexus of mice and allowed to clot at room temperature for 4 h. After clotting, the samples were centrifuged at 3000 rpm for 15 min at 4 °C to obtain serum. Serum levels of alanine aminotransferase (ALT), aspartate aminotransferase (AST), urea (UREA), creatinine (CREA), creatine kinase (CK), creatine kinase-MB (CK-MB), urea (UREA), creatinine (CREA), lactate dehydrogenase (LDH) and lactate dehydrogenase 1 (LDH1) were measured using an automated biochemical analyzer (Chemray-420, Rayto, CHN).

### Statistical analysis

Data are presented as the mean ± standard deviation (SD) with all experiments performed in triplicate to ensure reproducibility. Statistical comparisons were carried out using Student’s t-test for pairwise comparisons and one-way analysis of variance (ANOVA) for multi-group comparisons. Statistical significance was determined at the threshold of *P* < 0.05, with the following markers indicating the levels of significance: * for *P* < 0.05, ** for *P* < 0.01, and *** for *P* < 0.001.

## Results

### Preparation and characterization of DOX@PGA-PEG-GA NDs

PGA-PEG-GA was synthesized through a multi-step procedure outlined in Scheme S1. The synthesis began with the preparation of GA-functionalized PEG (PEG-GA) via esterification between activated GA and PEG. Successful conjugation was confirmed by ¹H-NMR (Fig. S1A), evidenced by the complete disappearance of the carboxylic acid proton signal of GA (δ = 12.2 ppm) and the emergence of characteristic PEG peaks (δ = 3.5 ppm) [41, 42]. Complementary FT-IR analysis (Fig. S1B) confirmed the retention of GA’s structure including hydroxyl (-OH, 3452 cm⁻¹) and carbonyl stretching vibrations (C = O, 1656 cm⁻¹), alongside the distinctive C-O-C skeletal vibrations of PEG (1105 cm⁻¹). The emergence of a new ester carbonyl peak at 1726 cm^−1^ provided direct evidence of ester bond formation. UV-Vis analysis (Fig. S1C) further validated the structural integrity of PEG-GA, which retained the characteristic absorption peak of GA at λ_max_ = 261 nm, indicating minimal electronic perturbation during conjugation.

**Fig. 2:**
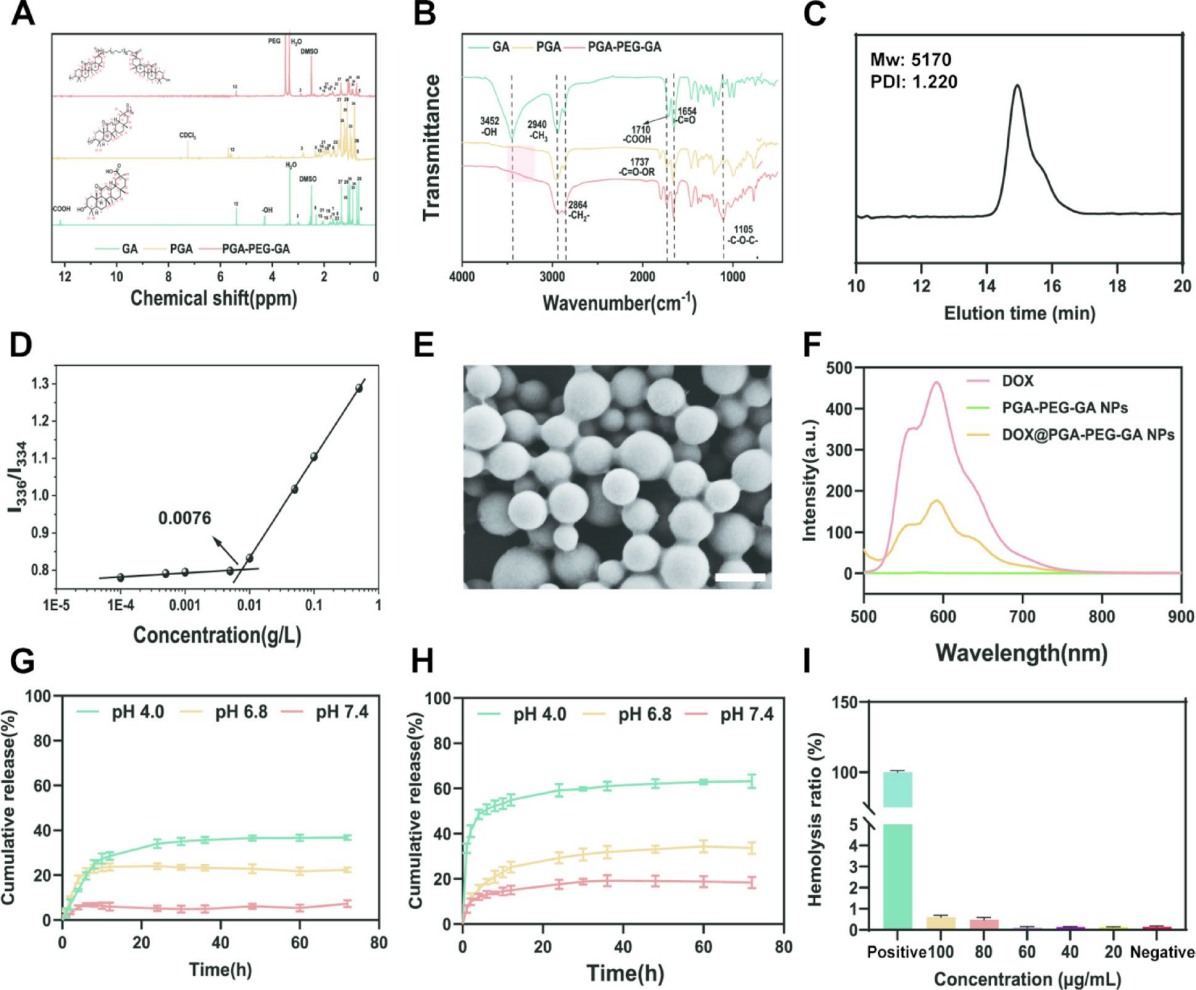
Characterization of PGA-PEG-GA Copolymer and Its Nanodrugs. (**A**) ^1^H-NMR spectra of GA, PGA, and PGA-PEG-GA. (**B**) FT-IR spectra of GA, PGA, and PGA-PEG-GA. (**C**) GPC profile of PGA-PEG-GA. (**D**) CMC of PGA-PEG-GA. (**E**) SEM images of DOX@PGA-PEG-GA NDs. Scale bar: 100 nm. (**F**) Fluorescence spectra of DOX, PGA-PEG-GA NDs, and DOX@PGA-PEG-GA NDs (excitation at 480 nm). (**G-H**) In vitro DOX release from DOX@PGA-PEG-GA NDs at various pH values: **G**) without esterase and **H**) with esterase. (**I**) In vitro hemolysis assay of DOX@PGA-PEG-GA NDs. Data are presented as mean ± SD (*n* = 3)

PGA-PEG-GA was subsequently synthesized by conjugating PEG-GA to the PGA backbone *via* an esterification reaction. The synthesis of PGA was first systematically optimized by examining the effects of reaction time and feed ratio on the molecular weight (Mw), polydispersity index (PDI), and degree of polymerization (DP). As summarized in Tables S1 and S2, a reaction time of 0.5 h with a GA to SOCl₂ molar ratio of 1:2 afforded PGA with an optimal balance of molecular weight (Mw = 2843 g/mol) and polydispersity index (PDI = 1.04), ensuring reproducible PGA formation. The successful conjugation of PEG-GA to PGA was verified by ¹H-NMR (Fig. 2A). While the characteristic methyl and methylene proton signals of PGA (0.8–1.5 ppm) remained unchanged, significant attenuation of the hydroxyl (4.3 ppm) and carboxyl (12.2 ppm) peaks indicated their consumption during polymerization. The appearance of characteristic PEG signals at 3.5 ppm, alongside retained GA and PGA resonance, confirmed covalent grafting. Further evidence came from FT-IR analysis (Fig. 2B), which showed the disappearance of PGA hydroxyl (-OH, 3452 cm⁻¹) and carboxylic acid (-COOH, 1710 cm⁻¹), and the emergence of a new ester carbonyl band at 1737 cm⁻¹. Additionally, the methylene (-CH₂-) peak at 2864 cm⁻¹ and the appearance of C-O-C stretching vibrations at 1105 cm⁻¹ confirmed PEG incorporation, while broadened peaks centered at 3300 cm⁻¹ corresponded to inter-molecular hydrogen bonding within PEG domains. These systematic spectra collectively corroborated the successful formation of PGA-PEG-GA. The controlled synthesis was further validated by complementary analytical techniques. UV-Vis spectroscopy (Fig. S2) revealed a bathochromic shift (λ_max_ = 264 nm) in PGA-PEG-GA compared to its precursors. Gel permeation chromatography (Fig. 2C) confirmed an increased molecular weight of 5170 g/mol with a PDI of 1.22, while X-ray diffraction (Fig. S3) illustrated a transition from crystalline (GA) to amorphous states (PGA and PGA-PEG-GA). The drug-loading potential of PGA-PEG-GA was demonstrated by pyrene fluorescence probe assays (Figs. 2D and S4), which revealed an ultralow critical micelle concentration (CMC = 0.0076 g/L), which is comparable to that of FDA-approved PCL-PEG formulation [43, 44]. The exceptionally low CMC indicates high stability against premature dissociation and underscores the potential of PGA-PEG-GA as a robust platform for nanoscale drug delivery [45].

Leveraging the exceptionally low CMC of PGA-PEG-GA, DOX was employed as a model drug to evaluate its drug loading and self-assembly properties. DOX-loaded nanodrugs (DOX@PGA-PEG-GA NDs) were prepared using the nanoprecipitation method and thoroughly characterized. Scanning electron microscopy (SEM, Fig. 2E) revealed monodisperse spherical nanoparticles with smooth surfaces and an average diameter of 85.8 ± 8.9 nm, slightly smaller than the hydrodynamic diameter measured by dynamic light scattering (Fig. S5A). Following DOX loading, the nanodrugs retained a negative zeta potential of −15.17 ± 0.12 mV, which is a key feature for prolonged blood circulation (Fig. S5B). Fluorescence spectroscopy (Fig. 2F) confirmed successful DOX encapsulation indicated by a characteristic emission peak at 600 nm (λ_ex_ = 480 nm). Additional spectroscopic evidence included the presence of DOX-specific FT-IR signatures (aromatic skeletal band at 1519 cm^−1^, Fig. S6A) and a UV-Vis absorption peak at 480 nm (Fig. S6B) [46]. As summarized in Table S3, the DOX@PGA-PEG-GA NDs exhibited a high drug loading capacity (8.82 ± 0.09%) and encapsulation efficiency (96.62 ± 0.93%).

The DOX@PGA-PEG-GA NDs displayed pH- and esterase-dependent release behavior. In vitro release studies (Fig. 2G-H) showed minimal DOX release (< 8%) at physiological pH 7.4, which increased markedly to 65% under acidic esterase-rich conditions (pH 4.0) mimicking the tumor microenvironment. In contrast, GA release was enhanced at neutral pH in the presence of esterase (Fig. S7). Hemocompatibility assays (Fig. 2I and S8) indicated negligible hemolysis (<1%) at 100 µg/mL. The structural integrity and systemic stability of the nanodrug are facilitated by PEG-mediated RES stealth properties and active targeting, promoting accumulation at tumor site [47]. The PEG-modified nanodrugs also showed enhanced colloidal stability compared to unmodified PGA NDs (Fig. S9). Furthermore, DOX@PGA-PEG-GA NDs maintained consistent hydrodynamic sizes and zeta potentials over 7 days in various media, including PBS, DMEM, and 10% FBS-supplemented DMEM (Fig. S10), demonstrating excellent stability for biological applications.

#### Investigation on the Self-assembly mechanism of PGA-PEG-GA and DOX

**Fig. 3:**
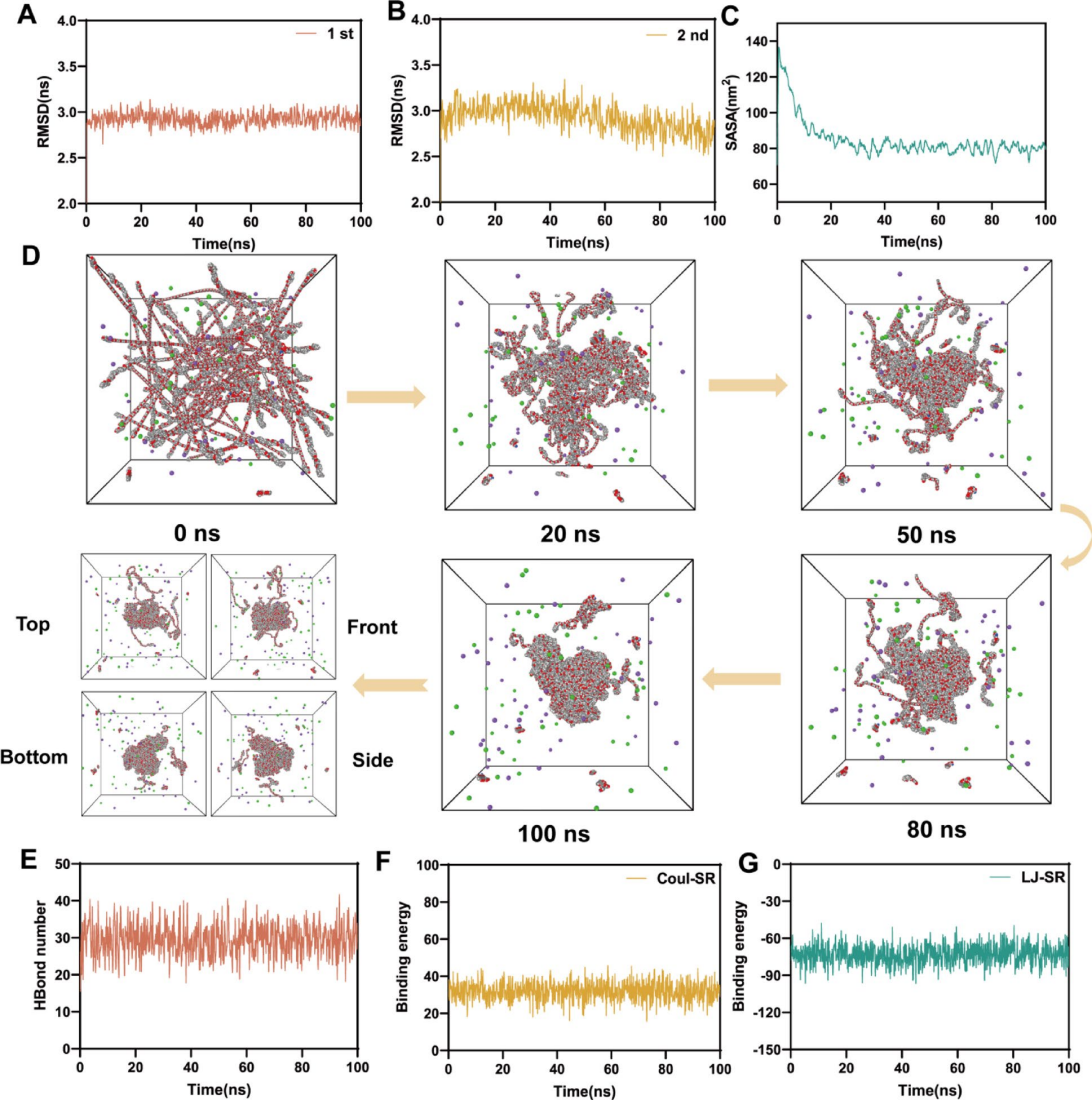
Molecular Dynamics Simulation of PGA-PEG-GA and DOX Interaction. (**A-B**) Root mean square deviation (RMSD) of PGA-PEG-GA during the (**A**) first and (**B**) second simulation periods. (**C**) Solvent-accessible surface area (SASA) of PGA-PEG-GA over the simulation time. (**D**) Snapshots of PGA-PEG-GA conformation at different simulation times (0 ns, 20 ns, 50 ns, 80 ns, 100 ns) from various perspectives (Top, Front, Bottom, Side). (**E**) Number of hydrogen bonds between PGA-PEG-GA and DOX. (**F**) Coulombic component of binding energy (Coul-SR) between PGA-PEG-GA and DOX. (**G**) Van der Waals component of binding energy (LJ-SR) between PGA-PEG-GA and DOX

Building upon the exceptionally low CMC and high DOX encapsulation capacity of PGA-PEG-GA, molecular dynamics (MD) simulations were employed to elucidate the self-assembly mechanism at the atomic level. Root Mean Square Deviation (RMSD) analyses (Fig. 3A-B) revealed converged trajectories with mean values of 3.02 ± 0.15 nm and 3.01 ± 0.13 nm, confirming structural stability and reproducibility of the simulation. Complementary Solvent Accessible Surface Area (SASA) calculations (Fig. 3C) showed a 46.4% decrease from 140 nm² to 75 ± 5 nm² over 100 ns, quantitatively supporting structural compaction driven by hydrophobic collapse. Sequential molecular snapshots (Fig. 3D, Supplementary Video S1) illustrated the dynamic self-organization process from initial molecular dispersion (0 ns) to well-defined spherical nanostructures (100 ns). Hydrogen bond analysis (Fig. 3E) indicated stabilization after 20 ns with an average of 31.2 ± 3.5 bonds, suggesting sustained intermolecular hydrogen bonding between PGA-PEG-GA and DOX.

Energy decomposition studies (Fig. 3F and G) identified van der Waals interactions (−78.4 ± 4.5 kJ/mol) as the dominant attractive force, surpassing electrostatic contributions by a 2.6-fold. Mechanistic validation via competitive binding assays (Fig. S11) showed NaCl (ionic strength modulator) and urea (hydrogen bond disruptor) had minimal impact on the hydrodynamic diameters of both blank and loaded nanodrugs, whereas Triton X-100 (a hydrophobicity inhibitor) induced significant disassembly [48]. Together, these multimodal results firmly establish that the self-assembly of DOX@PGA-PEG-GA NDs is governed by a synergistic mechanism dominated by hydrophobic and van der Waals interactions.

#### In vitro and in vivo targeting assays

**Fig. 4:**
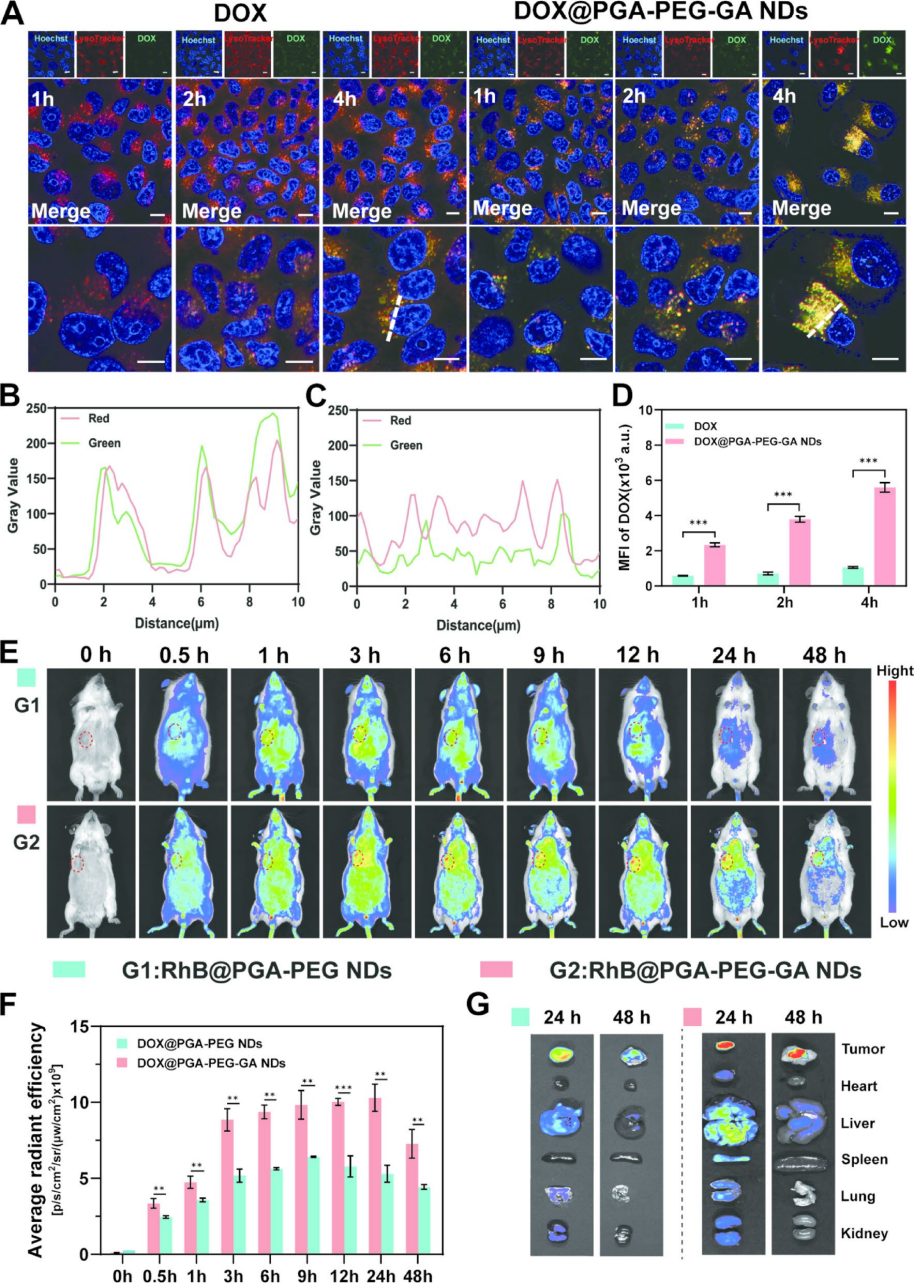
Targeted Drug Delivery and Biodistribution of Nanodrugs. (**A**) Confocal microscopy images of HepG2 cells treated with DOX and DOX@PGA-PEG-GA NDs at 1 h, 2 h, and 4 h. The cells were stained with DOX (green), LysoTracker (red), and Hoechst (blue). Scale bars: 10 μm. (**B-C**) Line scan analysis of the fluorescence intensity of green (DOX) and red (LysoTracker) channels in the confocal images with **B**) DOX and **C**) DOX@PGA-PEG-GA NDs. (**D**) Flow cytometric analysis of DOX fluorescence intensity in HepG2 cells treated with free DOX and DOX@PGA-PEG-GA NDs at 1 h, 2 h, and 4 h. (**E**) In vivo fluorescence imaging of mice administered with RhB@PGA-PEG NDs and RhB@PGA-PEG-GA NDs at various time points (0 h, 0.5 h, 1 h, 3 h, 6 h, 9 h, 12 h, 24 h, 48 h). (**F**) Time-dependent curve of fluorescence intensity at the tumor site. (**G**) Ex vivo fluorescence imaging of tumor and major organs (heart, liver, spleen, lung, kidney) from mice treated with RhB@PGA-PEG NDs and RhB@PGA-PEG-GA NDs at 24 h, and 48 h. Data are presented as mean ± SD (*n* = 3), with statistical significance indicated as *p < 0.05, **p < 0.01, *** *p* < 0.001

The spatiotemporal targeting precision of nanodrugs critically influences their therapeutic index by enhancing tumor-specific cytotoxicity through mechanisms such as receptor-mediated endocytosis (e.g., PKC targeting), while reducing off-target effects [49, 50]. To investigate the selective cellular uptake of DOX@PGA-PEG-GA NDs, cellular internalization in HepG2 and LX-2 cells was systematically evaluated using flow cytometry and confocal laser scanning microscopy (CLSM). Time-dependent quantification (Fig. S12) indicated significantly greater nanodrug uptake in HepG2 cells, with fluorescence intensities 2.4-fold higher at 1 h, 3.0-fold at 2 h, and 1.7-fold at 4 h compared to LX-2 cells. This preferential uptake was associated with overexpression of GA-specific receptors in HepG2 cells [51]. Quantitative colocalization analysis (Fig. 4A) further demonstrated strong lysosomal accumulation of DOX@PGA-PEG-GA NDs, supported by a high Pearson’s correlation coefficient (0.81 ± 0.05) with LysoTracker Red (Fig. 4B-C). In contrast, free DOX showed minimal lysosomal localization (Pearson’s coefficient = 0.26 ± 0.08), suggesting divergent internalization mechanisms between DOX@PGA-PEG-GA NDs and DOX. To determine whether cellular uptake was mediated by GA, a competitive inhibition assay was performed. As shown in Fig. S13, DOX@PGA-PEG-GA NDs displayed the highest fluorescence intensity, which was significantly greater than that of the DOX@PGA-PEG NDs group, indicating the enhanced delivery efficiency attributable to the PGA-PEG-GA. Furthermore, pre-treatment of HepG2 cells with free GA resulted in a marked reduction in the fluorescence intensity of DOX@PGA-PEG-GA NDs, confirming the essential role of GA in mediating targeted cellular internalization. The cellular uptake of H22 cells was further examined. As shown in Fig. S14, the cellular uptake of DOX@PGA-PEG-GA NDs by H22 cells was time-dependent. Fluorescence intensity in H22 cells was slightly higher than in HepG2 cells by 1.4-fold at 1 h, 1.4-fold at 2 h, and 1.6-fold at 4 h. Furthermore, when H22 cells were pre-inhibited with free GA, a marked reduction in fluorescence was observed compared to the untreated DOX@PGA-PEG-GA NDs group. This result further confirms the targeting role of GA, consistent with additional in vitro observations in HepG2 cells.

Sustained tumor retention is essential for effective drug accumulation and optimal therapeutic efficacy [52, 53]. To further study the role of GA-mediated active targeting in increasing the tumor accumulation of the nanodrug, we evaluated the in *vivo* biodistribution of RhB, RhB@PGA-PEG NDs, and RhB@PGA-PEG-GA NDs. As showed in Fig. 4E and G, both RhB@PGA-PEG NDs and RhB@PGA-PEG-GA NDs exhibited nearly identical biodistribution profiles, with peak accumulation at 9 h. However, at 24 h and 48 h, the tumor enrichment of RhB@PGA-PEG-GA NDs was 2.1-fold and 1.8-fold higher, respectively, compared to RhB@PGA-PEG ND. The ex vivo tissue enrichment results are consistent with the in vivo observation (Fig. S15). As shown in Fig. S16, free RhB accumulated rapidly in tumors, reaching a peak at 1 h postinjection, but was rapidly cleared with less than 10% of the signal remaining at 12 h. In contrast, the tumor enrichment of RhB@PGA-PEG-GA NDs was 18.3-fold higher than that of free RhB at 24 h. By 48 h, the RhB signal was negligible. These results strongly suggest that the enhanced permeability and retention (EPR) effect is the primary tumor-targeting mechanism [54]. This attenuated active targeting may be related to opsonin protein adsorption in the bloodstream, which likely inhibits GA-mediated active recognition. However, GA-modified nanodrugs retained significant tumor-targeting capability, allowing for more efficient accumulation at the tumor site, which holds promise for improving therapeutic outcomes.

## In vitro cytotoxicity assays

**Fig. 5:**
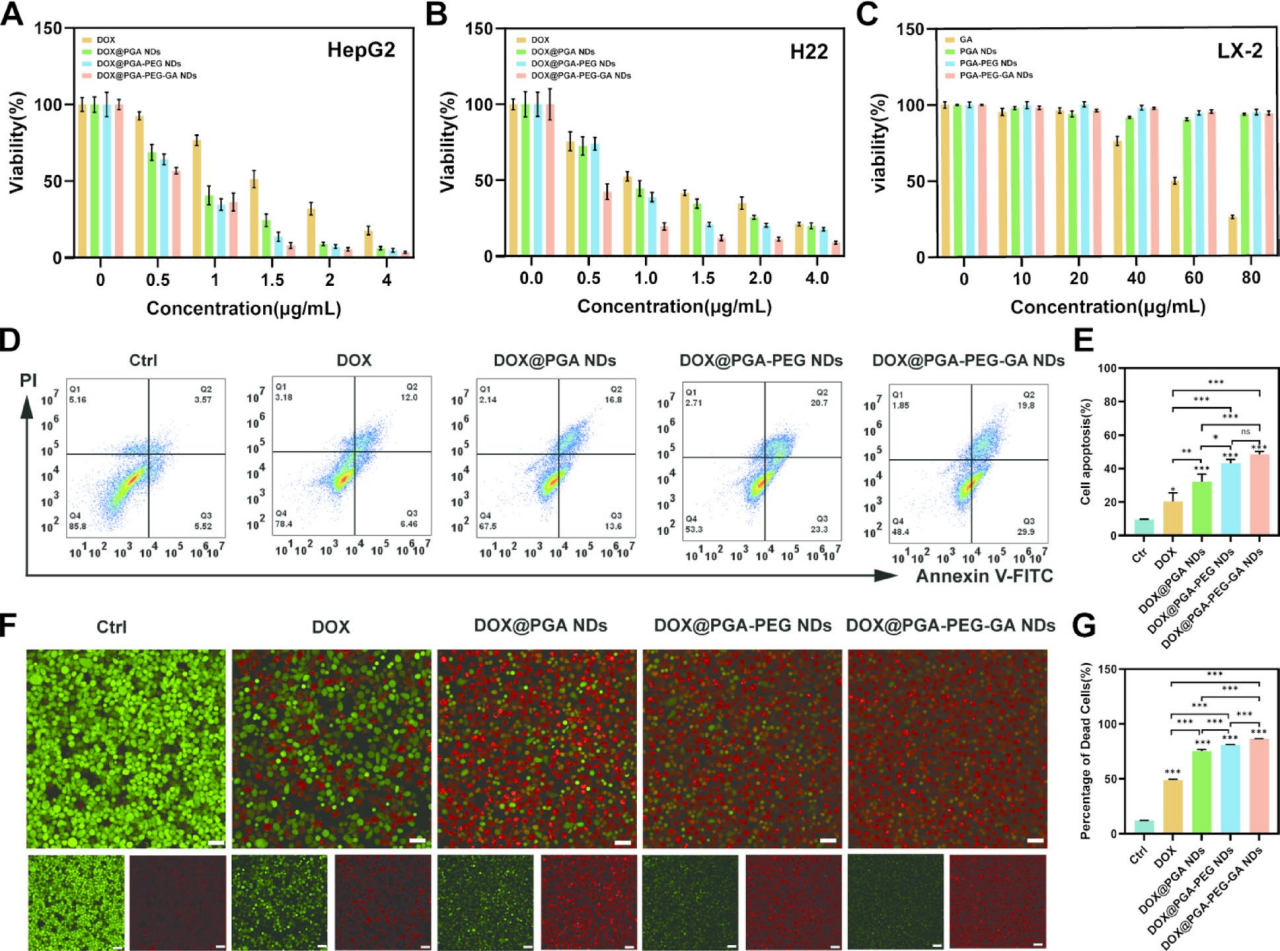
In Vitro Evaluation of Cytotoxicity, Apoptosis, and Dead Cell Analysis of Nanodrugs. (**A-B**) Cell viability of (**A**) HepG2 and (**B**) H22 cells treated with different formulations (DOX, DOX@PGA NDs, DOX@PGA-PEG NDs, DOX@PGA-PEG-GA NDs) at concentrations of 0, 0.5, 1, 2, and 4 µg/mL. (**C**) Cell viability of LX-2 cells treated with different blank nanodrugs (GA, PGA, PGA-PEG, PGA-PEG-GA) at concentrations of 0, 20, 40, 60, and 80 µg/mL. (**D**) Flow cytometric analysis of apoptosis in HepG2 cells treated with different formulations. The percentages of cells in each quadrant (Q1–Q4) are indicated, representing different apoptotic stages. (**E**) Quantitative analysis of cell apoptosis rates. (**F**) Confocal microscopy images of dead cells (red fluorescence) and live cells (green fluorescence) in HepG2 cells treated with different formulations. Scale bars: 100 μm. (**G**) Quantitative analysis of dead cell percentages. Data are presented as mean ± SD (*n* = 3), with statistical significance indicated as *p < 0.05, **p < 0.01, *** *p* < 0.001

A comprehensive in vitro cytotoxicity assessment of the synthesized nanodrugs was performed using CCK-8 assays, Annexin V-FITC/PI apoptosis analysis by flow cytometry, and live/dead cell staining. In HepG2 cells, blank nanodrugs (PGA NDs, PGA-PEG NDs, and PGA-PEG-GA NDs) exhibited dose-dependent cytotoxicity, showing 1.2-fold, 1.7-fold, and 1.9-fold greater potency compared to free GA at 80 µg/mL, respectively (Fig. S17A). DOX-loaded nanodrugs further enhanced antitumor activity with IC_50_ values of 0.78 ± 0.07 for DOX@PGA NDs, 0.68 ± 0.047 for DOX@PGA-PEG NDs, and 0.61 ± 0.084 µg/mL for DOX@PGA-PEG-GA NDs, corresponding to 2.0-fold, 2.3-fold, and 2.6-fold increases in potency over free DOX (Fig. 5A and Table S4). A similar trend was observed in H22 cells, where DOX@PGA-PEG-GA NDs showed maximal efficacy (IC_50_ = 0.38 ± 0.07 µg/mL), representing a 3.1-fold improvement compared to free DOX (Fig. S17B, Fig. 5B, and Table S4). In contrast, normal LX-2 cells maintained > 95% viability across all blank nanodrugs (0–80 µg/mL), demonstrating a safety profile comparable to free GA (Fig. 5C). Moreover, DOX-induced toxicity in LX-2 cells was significantly reduced by nano-formulation: DOX@PGA-PEG-GA NDs, DOX@PGA-PEG NDs, and DOX@PGA NDs reduced toxicity 23.9%, 40.0%, and 46.6%, respectively (Fig. S17C). The enhanced tumor-specific toxicity combined with reduced off-target effects underscores the potential of PGA-PEG-GA as a promising platform for precision oncology. Meanwhile, the relationship between GA loading efficiency and anti-tumor efficacy, as well as the synergistic therapeutic effect between DOX and PGA-PEG-GA, were investigated. As shown in Table S5, at an equivalent GA concentration, PGA NDs exhibited superior antitumor efficacy compared to free GA, which may be attributed to the nano-size effect of PGA NDs. Further functionalization to form PGA-PEG resulted in additional enhancement of antitumor activity, likely due to the improved stability of PGA-PEG NDs leading to increased cellular uptake. The introduction of active targeting further augmented the antitumor performance, which can be ascribed to GA receptor-mediated endocytosis. This observation is consistent with the results presented in Fig. 4. The combination index of DOX@PGA-PEG-GA NDs was also evaluated. The combination indices for H22 and HepG2 were 0.42 and 0.54, respectively. Both values are below 1, confirming that the combination of DOX and PGA-PEG-GA exhibits a strong synergistic effect.

To elucidate the mechanism underlying the enhanced cytotoxicity, apoptosis was quantitatively evaluated in HepG2 cells via Annexin V-FITC/PI dual staining and flow cytometry. At an equivalent GA concentration (20 µg/mL), PGA-PEG-GA NDs induced 2.97-fold higher apoptosis than free GA (13.47 ± 0.31% vs. 4.54 ± 0.13%), while PGA-PEG NDs (11.59 ± 0.41%) and PGA NDs (8.94 ± 0.48%) showed 2.55-fold and 1.97-fold increase, respectively (Figs. S18). This efficacy hierarchy (PGA-PEG-GA >PGA-PEG >PGA) correlated with structural enhancements promoting cellular internalization. At a therapeutically equivalent DOX concentration (1.5 µg/mL), all nanodrugs significantly enhanced apoptosis. Complementary live/dead staining using calcein-Amand propidium iodide (PI) further confirmed the cytotoxicity trend. Blank nanodrug showed progressively enhanced cytotoxicity with dead cell rates increasing from 13.34 ± 0.90% (free GA) to 15.02 ± 0.58% (PGA NDs), 23.49 ± 1.78% (PGA-PEG NDs) and 28.30 ± 2.24% (PGA-PEG-GA NDs) under the identical concentrations (20 µg/mL, Fig. S19). This graded response (PGA-PEG-GA >PGA-PEG >PGA) aligned with CCK-8 and apoptosis assays, underscoring the role of surface engineering in enhancing GA bioactivity. Under therapeutic equivalence conditions (1.5 µg/mL DOX), DOX@PGA-PEG-GA NDs induced 86.68 ± 0.22% cell death with 1.81-fold higher than free DOX (47.96 ± 2.39%) (Fig. 5F-G). Confocal microscopy revealed pronounced PI nuclear staining, loss of calcein-AM signal, and necrotic morphology such as membrane blebbing and cytoplasmic vacuolization. These results collectively demonstrate the dual advantage of the nanodrugs in enhancing therapeutic efficacy coupled with reduced off-target toxicity [55, 56].

## In vivo antitumor assay

**Fig. 6:**
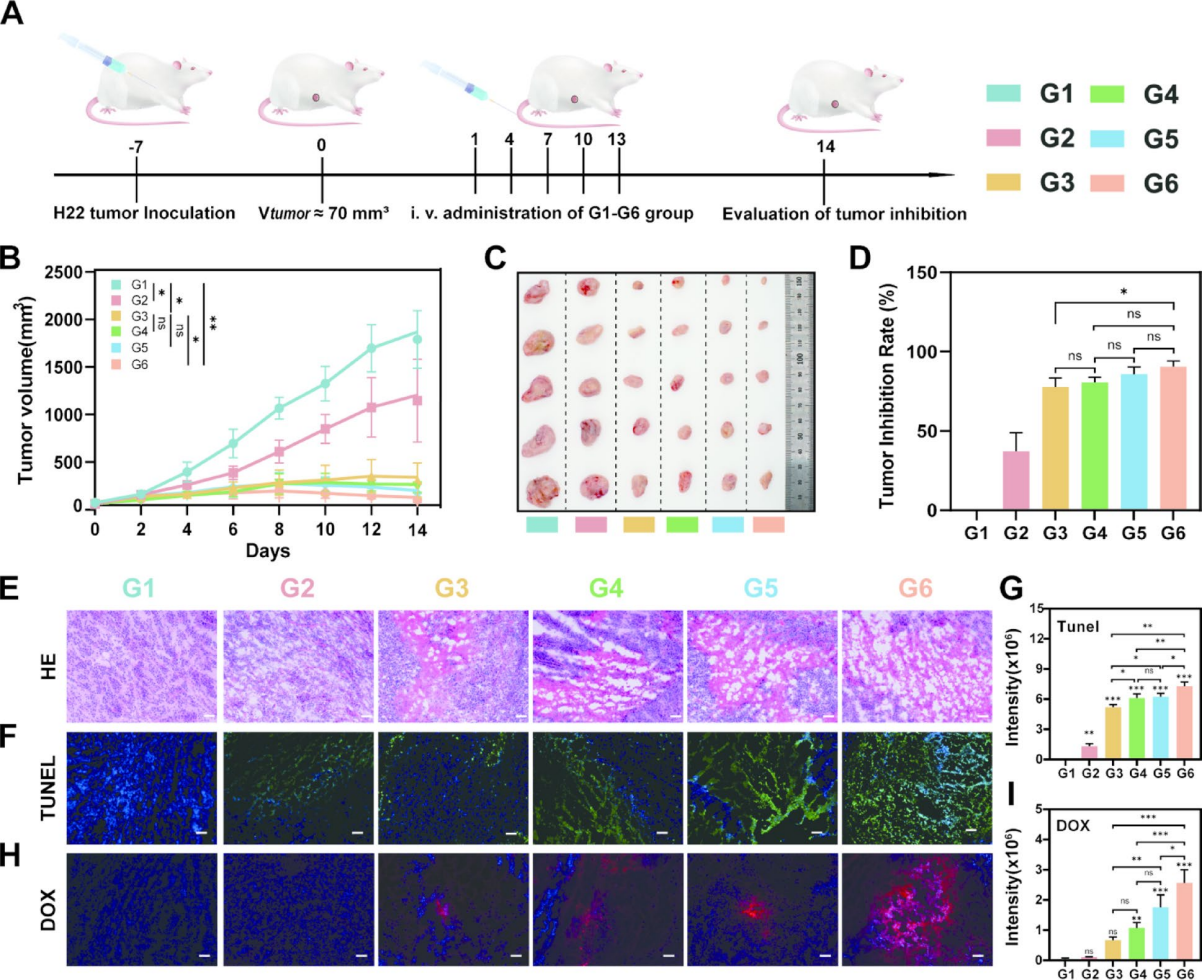
In Vivo Antitumor Efficacy and Histological Analysis of Nanodrugs. (**A**) Schematic timeline of the in vivo antitumor efficacy evaluation. H22 tumor-bearing mice were inoculated with tumor cells, and when the tumor volume reached approximately 70 mm³, different formulations (G1: Saline, G2: PGA-PEG-GA NDs, G3: DOX, G4: DOX@PGA NDs, G5: DOX@PGA-PEG NDs, G6: DOX@PGA-PEG-GA NDs) were intravenously administered. The tumor inhibition effect was assessed at day 14. (**B**) Tumor volume changes over time in different treatment groups (G1-G6). (**C**) Representative images of excised tumors from different treatment groups. (**D**) Tumor inhibition rate (%) calculated from the tumor weights of different groups. Data are presented as mean ± SD (*n* = 5), with statistical significance indicated as *p < 0.05, **p < 0.01, ***p < 0.001. (**E**) HE staining of different groups of tumor tissues, scale bar: 100 μm. (**F**) TUNEL staining of different groups of tumor tissues, scale bar: 100 μm. (**G**) Quantitative analysis of TUNEL fluorescence intensity. (**H**) DOX fluorescence of different groups of tumor tissues, scale bar: 100 μm. (**I**) Quantitative analysis of DOX fluorescence intensity. Data were expressed as mean ± SD (*n* = 3), and statistical significance was expressed as *p < 0.05, **p < 0.01, *** *p* < 0.001

Building on promising in vitro results, the in vivo therapeutic efficacy of the DOX@PGA-PEG-GA NDs was evaluated in H22 tumor-bearing Kunming mice. The mice were randomly divided into six groups (*n* = 5): Saline, PGA-PEG-GA NDs, free DOX, DOX@PGA NDs, DOX@PGA-PEG NDs, and DOX@PGA-PEG-GA NDs. As outlined in Fig. 6A, this multimodal design allowed for precise evaluation of each formulation’s antitumor activity. As demonstrated in Fig. 6B, C and S20, blank PGA-PEG-GA NDs alone showed significant intrinsic tumor growth inhibition (TGI = 40.09 ± 11.94%, vs. saline), which was consistent with in vitro observation. Among drug-loaded formulations, a hierarchical efficacy was evident. Free DOX achieved a TGI of 76.85 ± 11.00%, while DOX@PGA NDs, DOX@PGA-PEG NDs, and DOX@PGA-PEG-GA NDs showed 81.27 ± 5.05%, 85.10 ± 8.04%, and 89.81 ± 6.08%, respectively (Fig. 6D and S21). The enhanced efficacy is attributed to the pH and esterase-responsive release of DOX, active targeting via ligand-receptor interactions, and improved drug bioavailability. The superior performance of DOX@PGA-PEG-GA NDs underscores the importance of structural optimization in achieving maximal therapeutic effect through synchronized targeting and controlled release.

Histopathological analysis *via* terminal deoxynucleotidyl transferase dUTP nick-end labeling (TUNEL) and hematoxylin-eosin (HE) staining provided further mechanistic insight (Fig. 6E-G). HE staining revealed increasing levels of tumor cell damage, characterized by karyopyknosis, eosinophilic cytoplasm, and coagulative necrosis, correlating with therapeutic efficacy. TUNEL assays confirmed significantly higher apoptosis in the DOX@PGA-PEG-GA NDs group with quantitative analysis indicating that expanded necrotic areas (7.26 ± 0.45 × 10^6^) compared to free DOX (5.18 ± 0.27 × 10^6^). Furthermore, fluorescence imaging of cryosection showed a 4.3-fold higher DOX intensity in tumors treated with DOX@PGA-PEG-GA NDs than free DOX (Fig. 6H-I). These findings confirm that the nanodrug improves the therapeutic index through enhanced tumor targeting, subcellular trafficking, and activation of c apoptotic pathways.

Pharmacokinetic studies were conducted after intravenous administration of DOX@PGA-PEG-GA NDs (5 mg/kg DOX-equiv) and free DOX (5 mg/kg) in H22 tumor-bearing mice. Blood was collected over 48 h and DOX concentration was measured fluorometrically (λ_ex_/λ_em_ = 480/600 nm). As summarized in Fig. S22 and Table S6, the DOX@PGA-PEG-GA NDs exhibited significantly improved pharmacokinetic parameters. DOX@PGA-PEG-GA NDs demonstrated 3.0-fold longer mean residence time (MRT: 1.30 ± 0.23 h vs. 0.43 ± 0.06 h), 2.7-fold higher AUC (5.84 ± 0.64 vs. 2.13 ± 0.30 mg·h·L⁻¹), 2.9-fold extended elimination half-life (t_₁/₂_: 10.20 ± 0.50 h vs. 3.50 ± 0.39 h), and a 3.5-fold reduction in systemic clearance (CL: 0.02 ± 0.01 vs. 0.07 ± 0.01 L·h⁻¹), respectively. These enhancements are likely due to PEG-induced evasion of mononuclear phagocyte system uptake, as supported by reduced liver accumulation [57].

### In vivo biosafety evaluation

Systemic toxicity represents a major obstacle to the success of in vivo drug therapies, underscoring the necessity of rigorous safety monitoring [58]. To comprehensively evaluate the biosafety of the nanodrugs, physiological assessment and histopathological examinations were conducted. As shown in Fig. S23, no statistically significant weight loss was observed in any nanodrug-treated groups compared to the free DOX group. Free DOX administration resulted in significantly reduced hepatosplenic indices (liver: 4.83 ± 0.93% vs. 6.68 ± 1.12% in controls; spleen: 0.23 ± 0.05% vs. 0.70 ± 0.14%), whereas all nanodrug-treated groups maintained organ-to-body weight ratios within physiological ranges (Fig. S24). Histopathological analysis (Fig. S25) revealed pronounced organ-specific toxicities induced by free DOX, including cardiotoxicity (characterized by myofibrillar loss and disorganization), hepatotoxicity (evidenced by hepatocellular necrosis and sinusoidal dilation), and splenic immunotoxicity (marked by lymphoid depletion in white pulp). In contrast, tissue from nanodrug-treated groups retained normal ultrastructure integrity across all organs examined, showing histomorphology comparable to saline controls. Furthermore, serum ALT and AST levels in the free DOX group (ALT = 307 ± 65 U/L, AST = 626 ± 101 U/L) were markedly elevated 7.3-fold and 2.9-fold, respectively, higher than those in the saline group (ALT = 42 ± 1 U/L, AST = 215 ± 45 U/L), indicating substantial drug-induced hepatotoxicity (Fig. S26). The strong concordance between histopathological findings and serum biochemical profiles demonstrates that the nanodrug platform effectively optimizes the safety-efficacy balance of chemotherapy through rational nanoengineering [59, 60].

#### In vitro cardioprotective effects

**Fig. 7:**
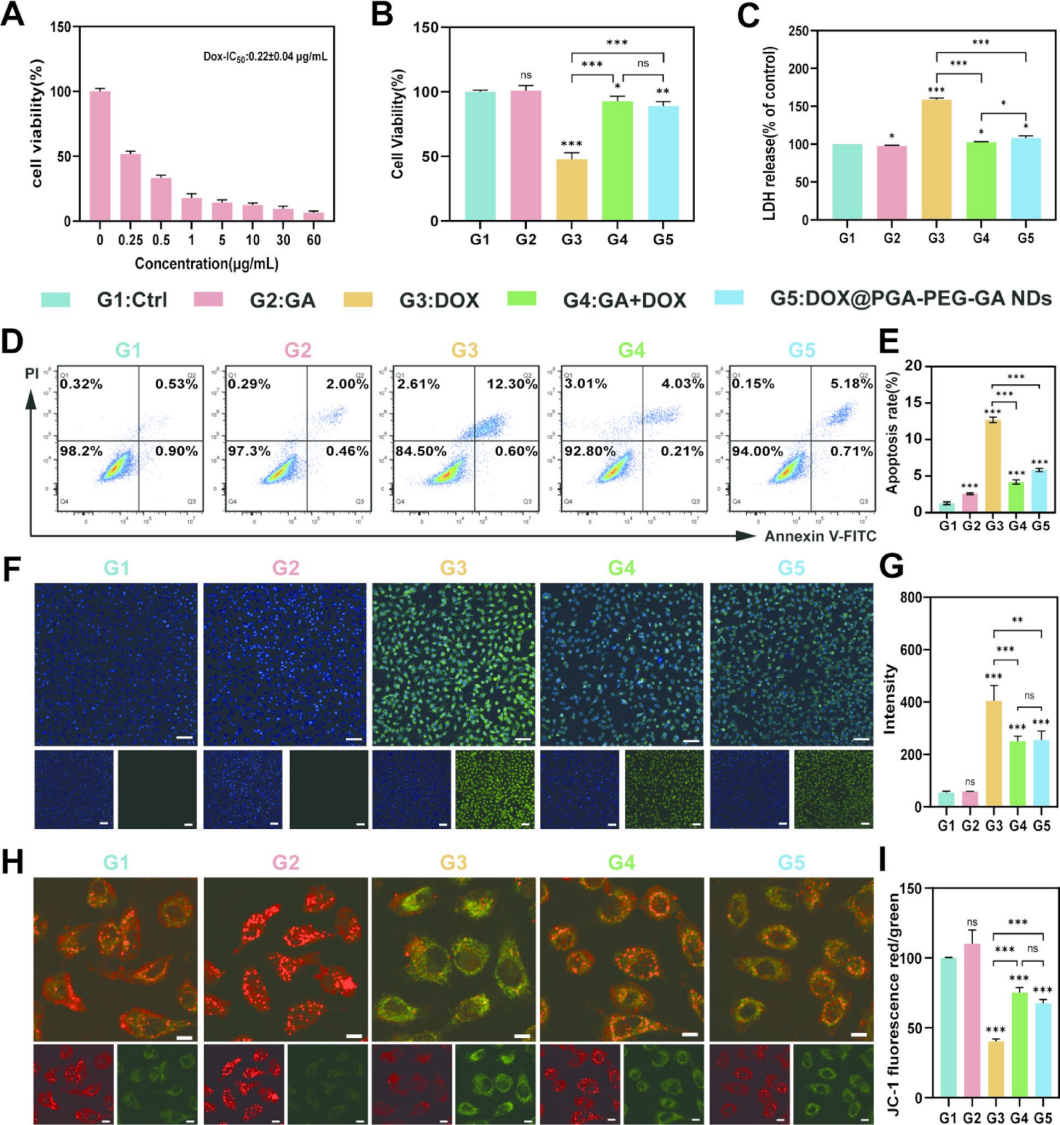
Protective Effects of GA and Its Nanocomposites on DOX-Induced Cytotoxicity in HCM Cells. (**A**) Cell viability of HCM cells treated with different concentrations of DOX. (**B-C**) Cell viability **B**) and LDH release **C**) of HCM cells treated with different formulations (G1: Ctrl, G2: GA, G3: DOX, G4: GA + DOX, and G5: DOX@PGA-PEG-GA NDs). (**D**) Flow cytometric analysis of apoptosis in HCM cells treated with different formulations. The percentage of cells in each quadrant (Q1-Q4) represents different apoptosis stages. (**E**) Quantitative analysis of apoptosis rates. (**F**) LCSM images of HCM cells treated with different formulations to observe ROS expression. Scale bar: 100 μm. (**G**) Quantitative analysis of green fluorescence intensity corresponding. (**H**) LCMS images of HCM cells treated with different formulations. Cells were stained with JC-1 (red/green) to observe changes in mitochondrial membrane potential. Scale bar: 10 μm. (**I**) Quantitative analysis of the JC-1 fluorescence ratio (red/green). Data are expressed as mean ± SD (*n* = 3), and statistical significance is indicated by *p < 0.05, **p < 0.01, *** *p* < 0.001

Although DOX remains a cornerstone chemotherapeutic agent, its clinic utility is substantially limited by dose-dependent cumulative cardiotoxicity—a critical side effect that severely restricts its therapeutic index [61, 62]. To investigate the cardioprotective mechanism of PGA-PEG-GA against DOX-induced cardiotoxicity, we systematically evaluated the effects of GA, free DOX, GA + DOX physical mixture, and DOX@PGA-PEG-GA NDs on human cardiomyocytes (HCM). As shown in Fig. 7A, CCK-8 assays confirmed pronounced DOX-induced cytotoxicity (IC_50_ = 0.22 ± 0.04 µg/mL). Notably, both the GA + DOX combination and DOX@PGA-PEG-GA NDs restored cell viability to near-normal levels, underscoring the cardioprotective role of GA and nanodrugs (Fig. 7B). Lactate dehydrogenase (LDH) release is a well-established indicator of loss of cellular membrane integrity. Assessment of membrane integrity via LDH release assays (Fig. 7C) revealed that free DOX increased LDH leakage by 1.6-fold compared to the control. In contrast, GA + DOX and DOX@PGA-PEG-GA NDs attenuated this leakage by 95.45 ± 1.27% and 86.59 ± 5.93%, respectively. Although GA itself induces minimal apoptosis, its release from PGA-PEG-GA is rapid only upon enzymatic cleavage by esterase at pH 7.4. Furthermore, the intracellular release rate of GA within HCM cells remains substantially below the threshold required to trigger apoptosis.

Apoptosis analysis further demonstrated that DOX@PGA-PEG-GA NDs reduced DOX-induced apoptosis by 2.2-fold (5.83 ± 0.22% vs. 12.69 ± 0.37% for free DOX), mirroring the protective effect observed with the GA + DOX combination (3.2-fold reduction) (Fig. 7D-E). Although GA itself induces minimal apoptosis, its release from PGA-PEG-GA is rapid only upon enzymatic cleavage by esterase at pH 7.4. Furthermore, the intracellular release rate of GA within HCM cells remains substantially below the threshold required to trigger apoptosis. ROS overproduction is a well-established central mechanism in DOX-induced cardiotoxicity [63, 64]. Using CLSM with the DCFH-DA probe, we confirmed significantly elevated ROS levels following DOX treatment (404.48 ± 58.27 vs. 54.14 ± 6.00 for control). Both GA + DOX and DOX@PGA-PEG-GA NDs significantly mitigated ROS generation, reducing levels by 38.1% and 36.9%, respectively (Fig. 7F-G). Evaluation of mitochondrial membrane potential (ΔΨm) via JC-1 ratiometry indicated that DOX caused severe depolarization, while GA + DOX and DOX@PGA-PEG-GA NDs markedly preserved ΔΨm (Fig. 7H-I). The red/green fluorescence ratio was restored 1.9-fold in the GA + DOX group and 1.7-fold in the DOX@PGA-PEG-GA NDs group compared to DOX treated cells (40.16 ± 1.80%). Collectively, these results indicate that DOX@PGA-PEG-GA NDs provide cardioprotection by reducing LDH release, apoptosis, ROS generation, mitochondria membrane potential loss, which is consistent with previously reported studies [33].

### In vivo cardioprotective effects

**Fig. 8:**
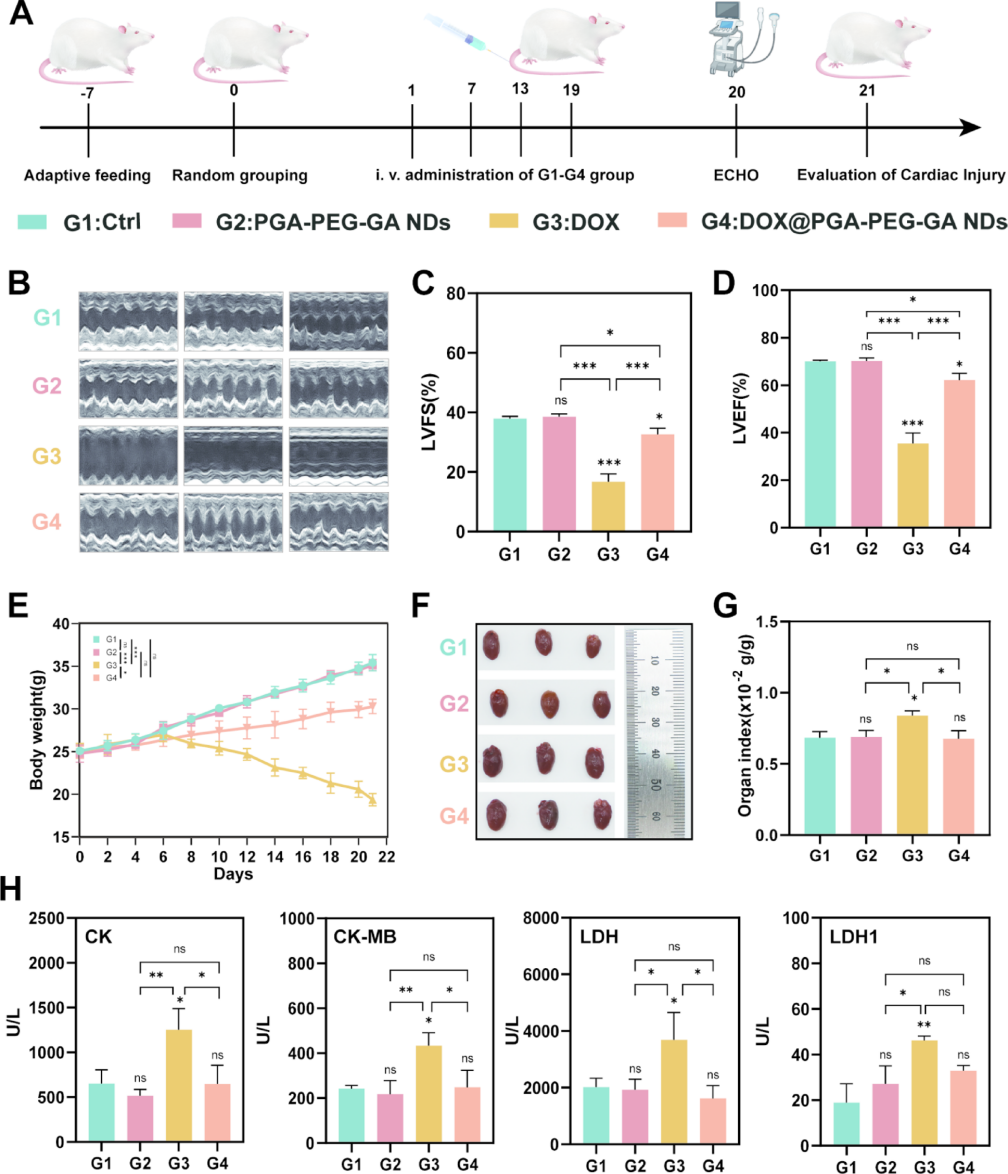
In Vivo Cardiac Safety Evaluation of Nanodrugs. (**A**) Schematic timeline of the in vivo cardiac safety evaluation. Mice were adaptively fed for 7 days, randomly grouped, and then intravenously administered with different formulations (G1: Saline, G2: PGA-PEG-GA NDs, G3: DOX, G4: DOX@PGA-PEG-GA NDs). Cardiac function was assessed by ECHO at day 20, and cardiac injury was evaluated at day 21. (**B**) Echocardiographic images of mice from different groups (G1-G4). (**C-D**) Quantitative analysis of left ventricular fractional shortening (LVFS, C) and left ventricular ejection fraction (LVEF, D) from echocardiographic images. (**E**) Body weight changes over time in different treatment groups (G1-G4). (**F**) Images of heart samples from different groups. (**G**) Heart weight to body weight ratio in different groups. (H) Levels of cardiac injury markers (CK, CK-MB, LDH, LDH1) in different groups. Data are presented as mean ± SD (*n* = 3), with statistical significance indicated as *p < 0.05, **p < 0.01, *** *p* < 0.001

To comprehensively evaluate the cardioprotective efficacy of DOX@PGA-PEG-GA NDs against DOX-induced cardiotoxicity in vivo, a systematic study was conducted in Kunming mice following the experimental scheme outlined in Fig. 8A. Echocardiographic (ECHO) analysis revealed pronounced intergroup functional disparities (Fig.8B). Echocardiographic (ECHO) analysis revealed pronounced differences in cardiac function across treatment groups (Fig. 8C-D). G1 and G2 exhibited normal cardiac function, with left ventricular ejection fraction (LVEF) values of 69.80 ± 0.80% and 70.23 ± 1.19%, and left ventricular fractional shortening (LVFS) values of 37.87 ± 0.85% and 38.53 ± 0.96%, respectively. In stark contrast, G3 displayed severe cardiac impairment with LVEF and LVFS values reduced to 16.70 ± 2.68% and 35.37 ± 4.48%, respectively. Remarkably, G4 showed substantial functional recovery with a 1.8-fold increase in LVEF (62.10 ± 2.86%) and a 2.0-fold improvement in LVFS (32.63 ± 2.08%) compared to G3, bringing these parameters close to those observed in the control groups (G1/G2).

Body weight changes also reflected treatment-related effects. As displayed in Fig. 8E, mice in G1 and G2 maintained physiological weight gain throughout the study. In contrast, G3 exhibited progressive weight loss beginning after the second administration (Day 7), culminating in a total loss of 21.97 ± 4.43%. Mice in G4, however, sustained healthy weight gain comparable to that of the control groups. Terminal gross pathological examination revealed marked cardiomegaly and ventricular dilation in G3 (Fig. 8 F–G), whereas G4 showed effective prevention of DOX-induced pathological hypertrophy with heart indices normalized to control levels. Quantitative serum biomarker analysis further highlighted cardiotoxicity in G3, with significant elevations in creatine kinase (CK, 1245 ± 234 U/L), creatine kinase MB isoenzyme (CK-MB, 433 ± 58 U/L), lactate dehydrogenase (LDH, 3692 ± 966 U/L), and lactate dehydrogenase 1 (LDH1, 46 ± 2 U/L) (Fig. 8H). In contrast, biomarker levels in G4 were comparable to those in untreated groups (G1/G2).

Multiparametric cardiac tissue analysis in Fig. S27 corroborated these observations. Histopathological evaluation revealed severe myofibrillar disarray and sarcomeric disruption in G3. In comparison, G4 exhibited a 6.43-fold reduction in ROS generation (1.60 ± 0.43 × 10^5^ vs. 1.03 ± 0.28 × 10^6^ in G3), reaching levels similar to those in G1 and G2. Apoptotic cells fluorescence intensity was also significantly reduced in G4 by 6.56-fold (1.14 ± 0.38 × 10^4^ vs. 7.48 ± 1.58 × 10^4^ in G3), returning to baseline values. Collectively, these multimodal findings demonstrate that DOX@PGA-PEG-GA NDs ameliorate chemotherapy-induced cardiotoxicity primarily through suppression of oxidative stress and inhibition of apoptosis.

## Discussion

This study presents the design and validation of a novel “drug-as-carrier” nanodrug, PGA-PEG-GA, which exploits the inherent pharmacological and targeting properties of GA to overcome key limitations of conventional nanocarriers. In contrast to traditional nanocarriers that often hampered by low drug loading, lack of intrinsic bioactivity, and passive release kinetics, our nanodrug achieves ultrahigh drug-loading capacity while enabling active targeting of HCC through GA-specific receptor recognition. The amphiphilic block copolymer self-assembles into stable micellar nanostructures with an exceptionally low CMC, promoting efficient drug encapsulation and sustained release of DOX in response to acidic and esterase-rich tumor microenvironments.

Our findings demonstrate that the structural and functional integration of GA into the polymer backbone significantly enhances cellular uptake in HCC cells, promotes superior tumor accumulation via both EPR and active targeting mechanisms, and elicits potent antitumor efficacy. Notably, the released GA acts synergistically with DOX not only to enhance antitumor efficacy but also to mitigate DOX-induced cardiotoxicity by reducing oxidative stress and mitochondrial dysfunction, as rigorously validated through in vitro and in vivo cardioprotective assays. Capitalizing on its amphiphilic nature, a double emulsion method is utilized to encapsulate FITC-labeled bovine serum albumin with a high encapsulation efficiency (84.26 ± 0.39%), significantly surpassing that of commercial PLGA-PEG nanoparticles (48.6 ± 1.1%) [65]. Furthermore, PGA-PEG-GA mitigates aseptic inflammation commonly associated with conventional polymeric material [66, 67]. Importantly, GA is FDA-recognized as safe, and PEG is well-established in clinical use. The copolymer is constructed through biodegradable ester linkages, ensuring complete breakdown into non-toxic metabolites, which is a critical feature supporting its clinical translation potential. Unlike conventional strategies that utilize GA solely as a targeting ligand, our work introduces a holistic “structure–function–safety” design that merges natural product pharmacology with polymer nanotechnology to create a multifunctional platform capable of simultaneous targeted therapy and organ protection. This integrated strategy effectively circumvents the excipient burden and functional fragmentation typical of conventional nanoformulations.

Despite these promising outcomes, further studies are warranted to elucidate the long-term toxicity, potential immune responses, and metabolic fate of PGA-PEG-GA degradation products. Additionally, scale-up synthesis and rigorous good laboratory practice toxicology studies will be critical to advance this platform toward clinical translation.

## Conclusion

In conclusion, this study successfully demonstrates the rational design and synthesis of a novel polymeric nanodrug, PGA-PEG-GA, leveraging GA as both a therapeutic monomer and an active targeting ligand for HCC. Our findings confirm the initial hypothesis that such a “drug-as-carrier” strategy can overcome fundamental limitations of conventional nanocarriers—achieving ultrahigh drug loading, intrinsic antitumor activity, and targeted delivery—while concurrently mitigating DOX-induced cardiotoxicity. The PGA-PEG-GA platform exhibited exceptional stability, pH/enzyme-responsive drug release, enhanced cellular uptake, and significant tumor accumulation, resulting in superior antitumor efficacy and reduced systemic toxicity. Notably, the nanodrug conferred cardioprotection via suppression of oxidative stress and apoptosis, underscoring its dual therapeutic and protective functions. These results represent a significant advancement in nanomedicine by integrating structural design, bioactivity, and safety into a single system. Moving forward, future work should focus on long-term in vivo toxicity, detailed mechanistic investigations into immune responses and metabolic pathways, and scale-up synthesis towards clinical translation. The versatility of this platform also warrants exploration for delivering other therapeutic agents (e.g., nucleic acids or immunomodulators), potentially broadening its application in precision oncology.

## Supplementary Information

Supplementary Material 1

Supplementary Material 2

## Data Availability

No datasets were generated or analysed during the current study.
