# Supplementary material for: Reengineering glycyrrhetinic acid into a therapeutic oligomer for targeted tumor therapy with cardioprotection
Source: J Nanobiotechnology. 2025 Nov 12;23:715. doi: 10.1186/s12951-025-03765-5 (PMC12613581; doi:10.1186/s12951-025-03765-5)
Supplement: Supplementary file 1 — Supplementary Material 1 [file 12951_2025_3765_MOESM1_ESM.docx]

**Supplementary material**

Reengineering Glycyrrhetinic Acid into a Therapeutic Oligomer for Targeted Tumor Therapy with Cardioprotection

*Zixin Wang ^a,1^,Bo Su ^a,1^, Alu Ouyang ^a,1^,ZiXuan Liang ^c^ ,Pingyun Yuan ^d^, Xin Qin ^a^, Yu Li ^a^ , Xuejing Huang ^a^，Ling Fan ^b^***, Hongwei Guo ^a^*, Ronghua Jin ^a^**

^a^ Guangxi Key Laboratory of Bioactive Molecules Research and Evaluation, Pharmaceutical College, Guangxi Medical University, 22 Shuangyong Road, Nanning, 530021, P R China

^b^ Key laboratory of Chemistry and Engineering of Forest Products, State Ethnic Affairs Commission, Guangxi Key Laboratory of Chemistry and Engineering of Forest Products, Guangxi Collaborative Innovation Center for Chemistry and Engineering of Forest Products, School of chemistry and chemical engineering, Guangxi Minzu University, Nanning, 530006, P R China.

^c^ Guangxi Medical University Cancer Hospital, Guangxi Medical University. No. 71 Hedi Road, Nanning, 530021, P R China

^d^ Shaanxi Key Laboratory of Biomedical Metal Materials, Northwest Institute for Nonferrous Metal

Research, Xi’an 710016, P. R. China

^1^ These authors contributed equally to this work.

*Corresponding authors:

Dr. Ronghua Jin, Guangxi Key Laboratory of Bioactive Molecules Research and Evaluation & Pharmaceutical College, Guangxi Medical University, 22 Shuangyong Road, Nanning 530021, P.R. China. E-mail address: [jinronghua@gxmu.edu.cn](mailto:jinronghua@gxmu.edu.cn)

Number of pages: 18

Number of figures: 24

Number of tables: 5

**Supplementary Figures**

**Scheme S1.** Synthetic routes to PGA-PEG-GA.

**Figure S1.** Characterization of PEG-GA.

**Figure S2.** UV-Vis absorption spectra of GA, PGA, and PGA-PEG-GA.

**Figure S3.** XRD patterns of GA, PGA, PEG, and PGA-PEG-GA.

**Figure S4.** Fluorescence excitation spectra of pyrene in PGA-PEG-GA micelles.

**Figure S5.** FT-IR and UV-Vis characterization of DOX@PGA-PEG-GA NDs.

**Figure S6.** Hydrodynamic diameter and zeta potential of PGA-PEG-GA NDs and DOX@PGA-PEG-GA NDs.

**Figure S7.** *In vitro* GA release profiles from DOX@PGA-PEG-GA NDs.

**Figure S8.** Hemolysis assay of DOX@PGA-PEG-GA NDs.

**Figure S9.** Stability analysis of PGA and PGA-PEG-GA NDs.

**Figure S10.** Colloidal stability of DOX@PGA-PEG-GA NDs in physiological media.

**Figure S11.** Self-assembly mechanism of PGA-PEG-GA NDs and DOX@PGA-PEG-GA NDs.

**Figure S12.** Flow cytometry analysis of DOX@PGA-PEG-GA NDs uptake in L-02 and HepG2 cells.

**Figure S13.** *Ex vivo* biodistribution of RhB@PGA-PEG-GA NDs.

**Figure S14.** Cytotoxicity of blank and DOX-loaded nanodrugs.

**Figure S15.** Apoptosis analysis of HepG2 cells by flow cytometry.

**Figure S16.** Live/dead cell staining of HepG2 cells.

**Figure S17.** Tumor growth curves in different treatment groups.

**Figure S18.** Quantitative tumor weight assessment.

**Figure S19.** Pharmacokinetic profiles of free DOX and DOX@PGA-PEG-GA NDs.

**Figure S20.** Body weight changes in mice during treatment.

**Figure S21.** Organ coefficients of mice post-treatment.

**Figure S22.** HE staining of major organs.

**Figure S23.** Serum biochemical indices of treated mice.

**Figure S24.** Histological analysis of heart tissues.

**Supplementary Tables**

**Table S1.** Effects of reaction time on PGA polymerization.

**Table S2.** Effects of feed ratios on PGA polymerization.

**Table S3.** Encapsulation efficiency and drug-loading capacity of NDs.

**Table S4.** IC_50_ values of drug treatments on HepG2, H22, and L-02 cells.

**Table S5.** Pharmacokinetic parameters of free DOX and DOX@PGA-PEG-GA NDs.


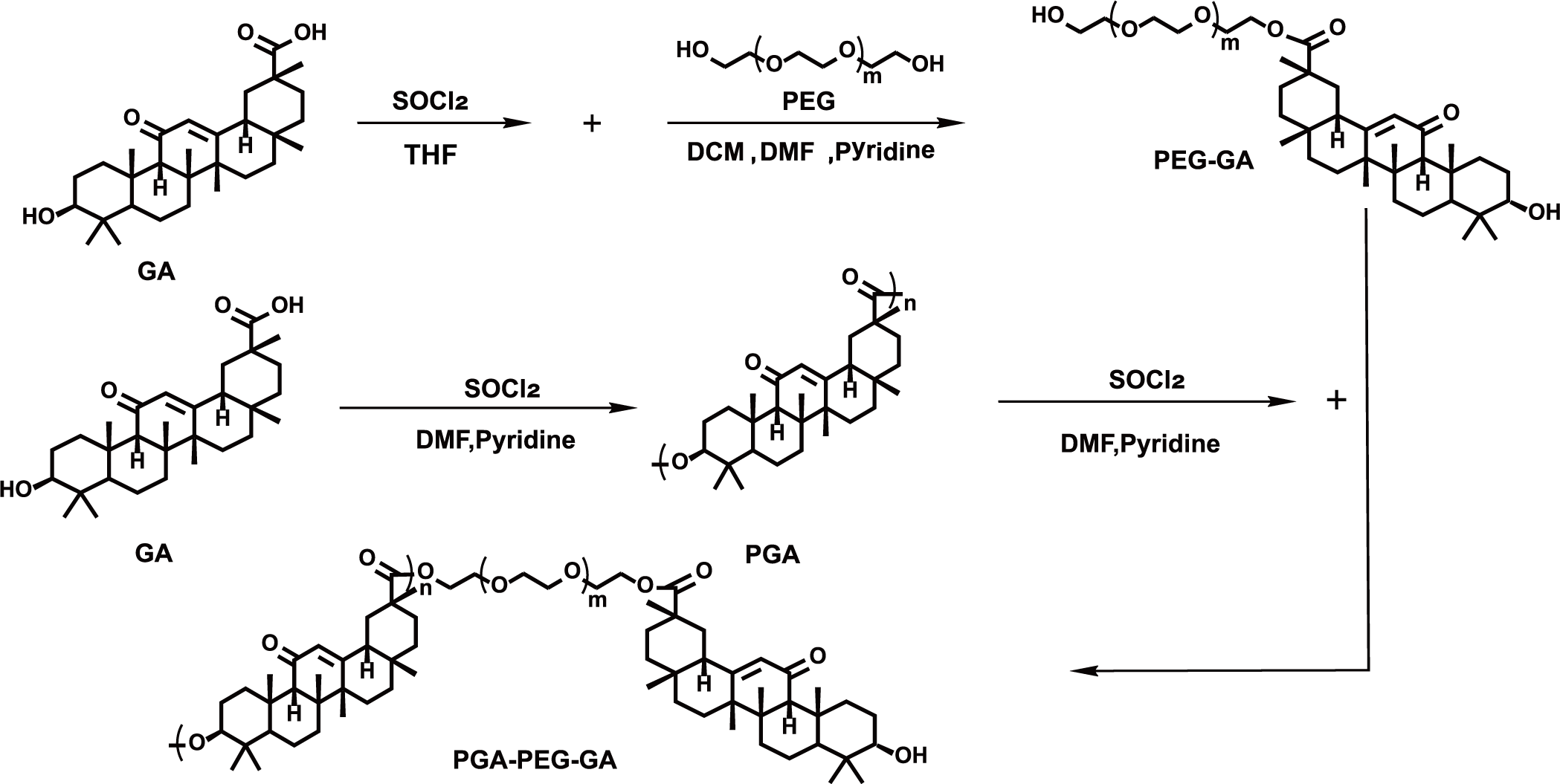


**Scheme S1**. The synthetic routes to PGA-PEG-GA.

**Table S1**. Effects of reaction time on the molecular weight, polymer dispersity index (PDI), and degree of polymerization (DP)

| Sample | Mn | Mw | Mp | PDI | DP |
| --- | --- | --- | --- | --- | --- |
| PGA-0.5h | 2486 | 2540 | 2510 | 1.022 | 5 |
| PGA-1h | 2128 | 2362 | 2596 | 1.110 | 5 |
| PGA-2h | 2212 | 2355 | 2510 | 1.065 | 5 |
| PGA-4h | 2079 | 2264 | 2552 | 1.089 | 4 |

**Table S2**. Effects of feed ratios on molecular weight, polymer dispersity index (PDI), and degree of polymerization (DP)

| Sample | Mn | Mw | Mp | PDI | DP |
| --- | --- | --- | --- | --- | --- |
| PGA-2:1 | 1823 | 2133 | 2346 | 1.170 | 5 |
| PGA-1:1 | 2548 | 2630 | 2510 | 1.032 | 5 |
| PGA-1:2 | 2748 | 2843 | 2510 | 1.035 | 6 |
| PGA-1:4 | 1534 | 1596 | 1620 | 1.040 | 3 |


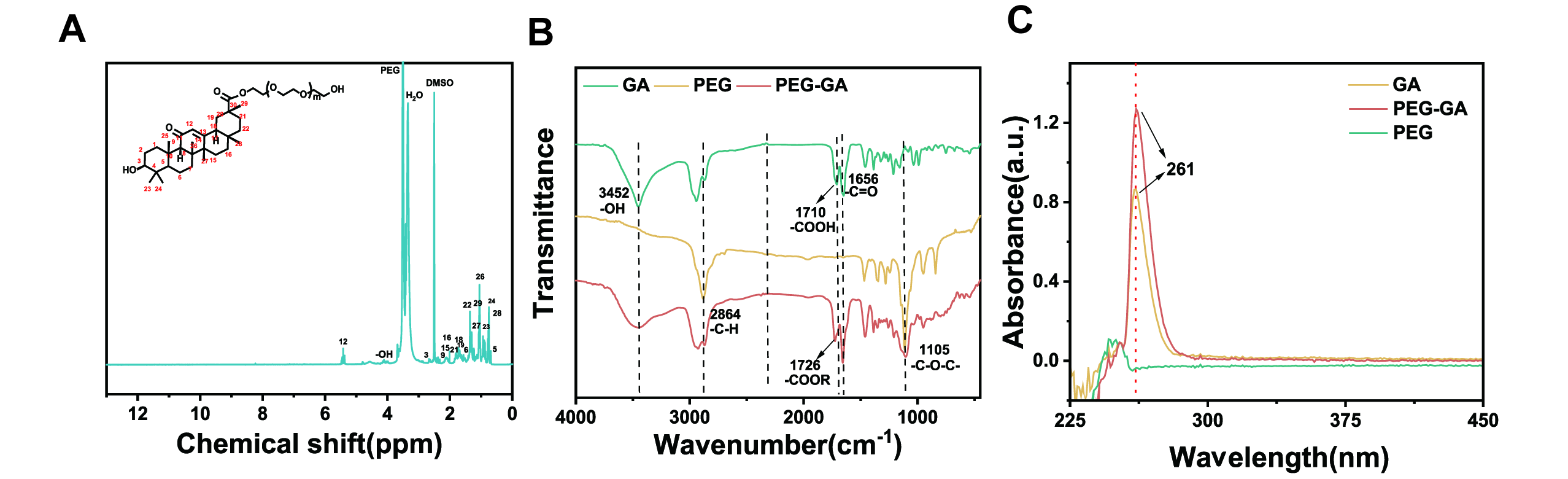


**Figure S1**. Characterization of PEG-GA. (A) ^1^H-NMR spectra, (B) FT-IR spectra, and (C) UV-Vis absorption spectra of GA, PEG, and PEG-GA.


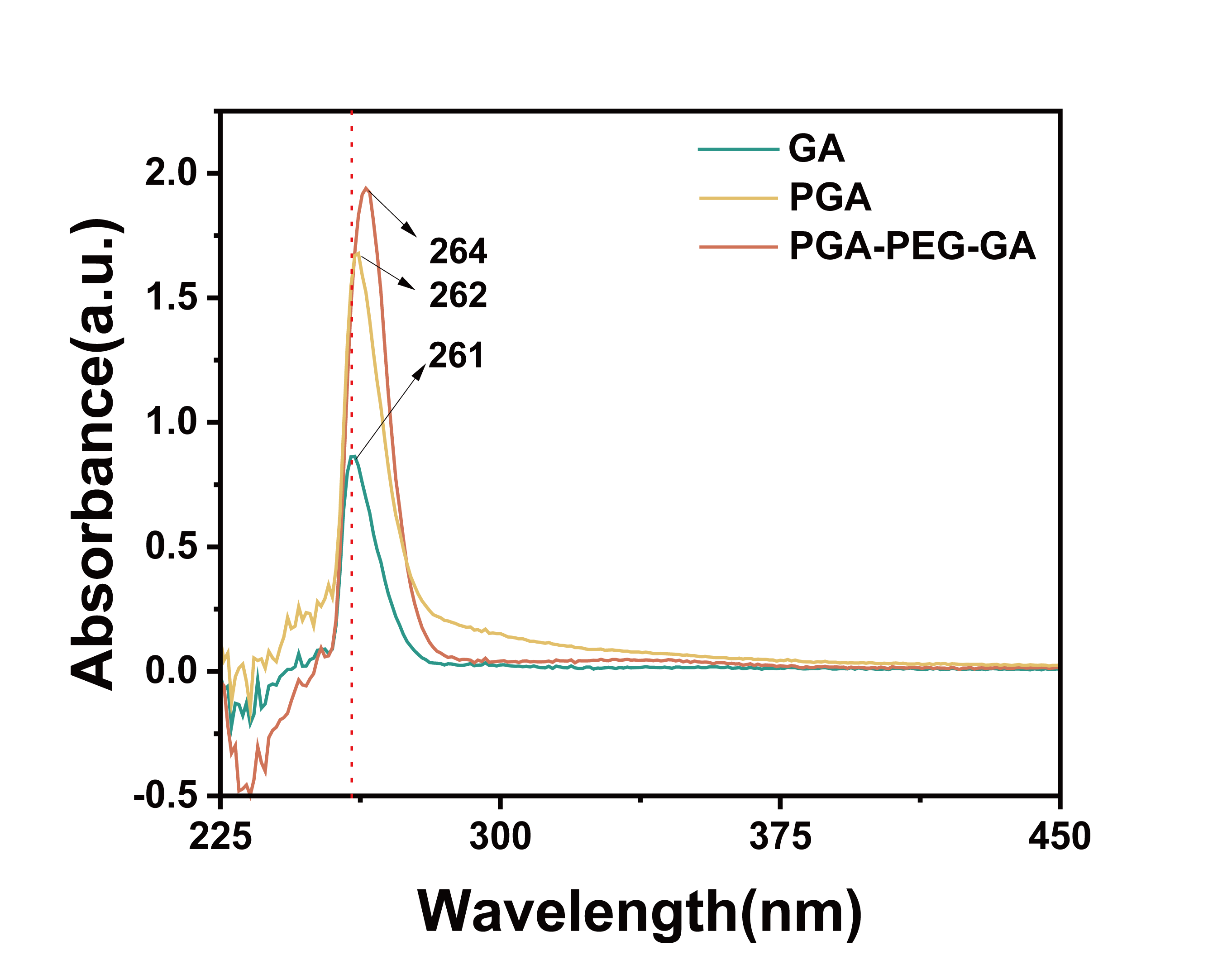


**Figure S2**. UV-Vis absorption spectra of GA, PGA, and PGA-PEG-GA.


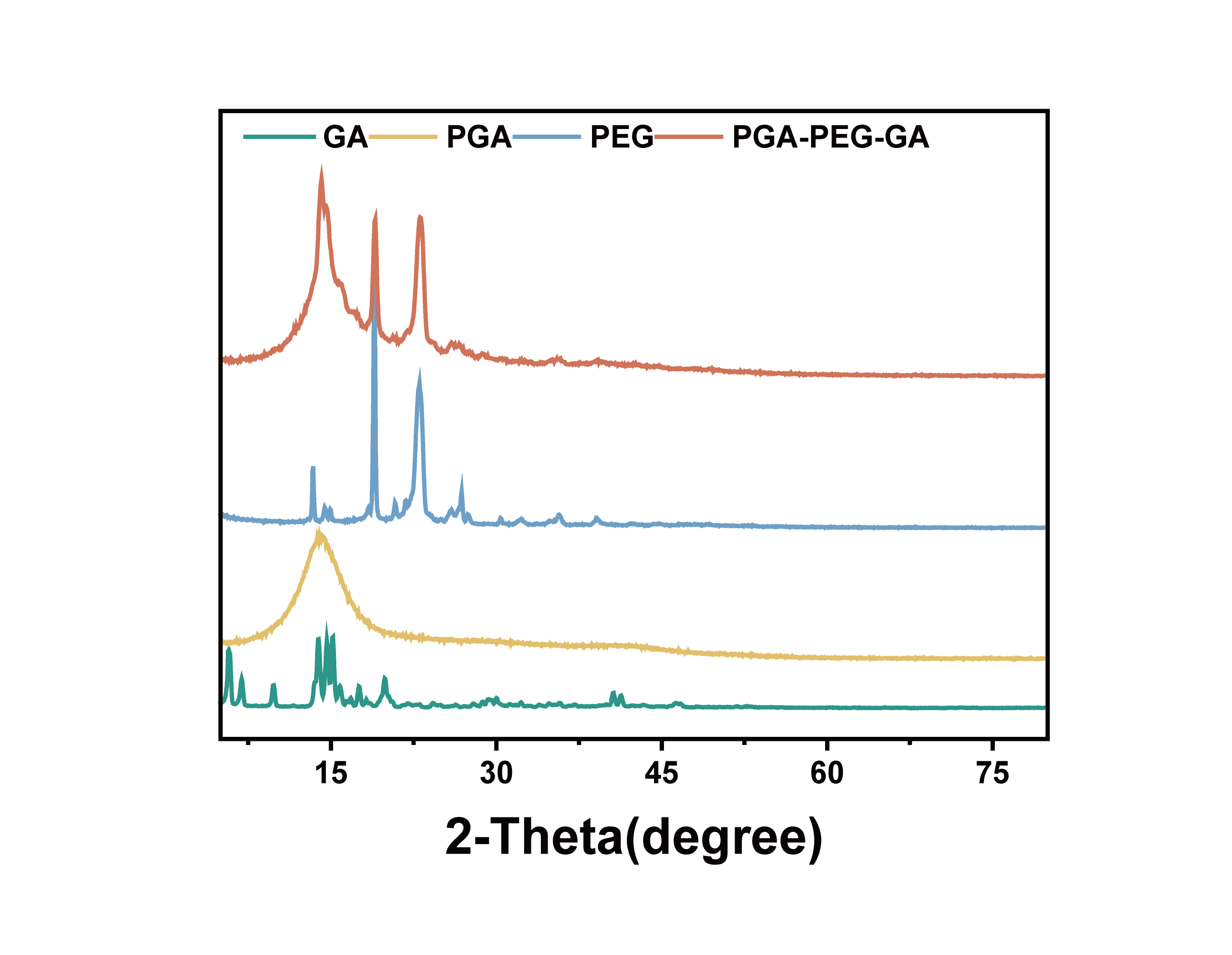


**Figure S3**. XRD patterns of GA, PGA, PEG, and PGA-PEG-GA.


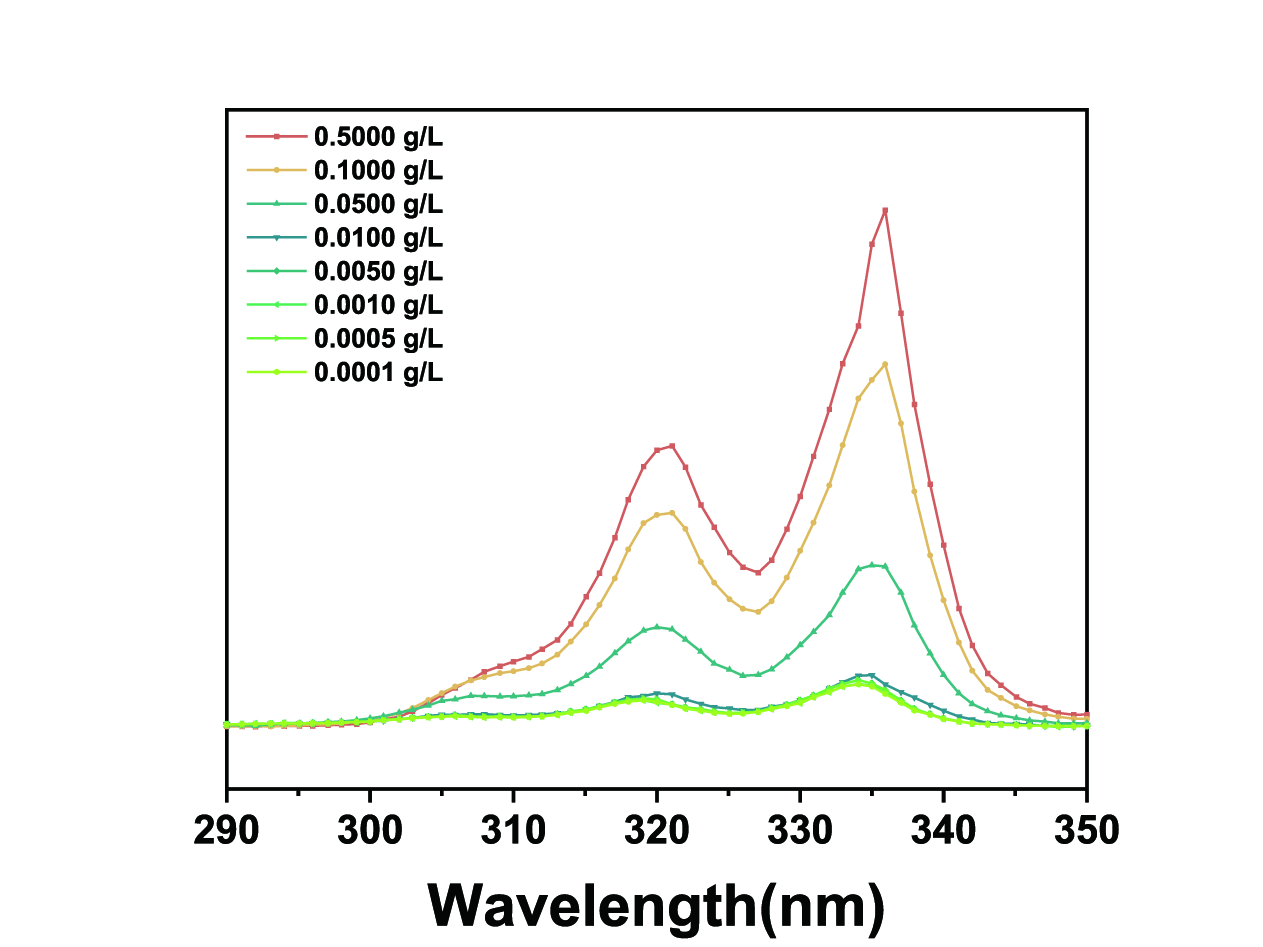


**Figure S4**. Fluorescence excitation spectra of pyrene co-incubated with PGA-PEG-GA micelles.


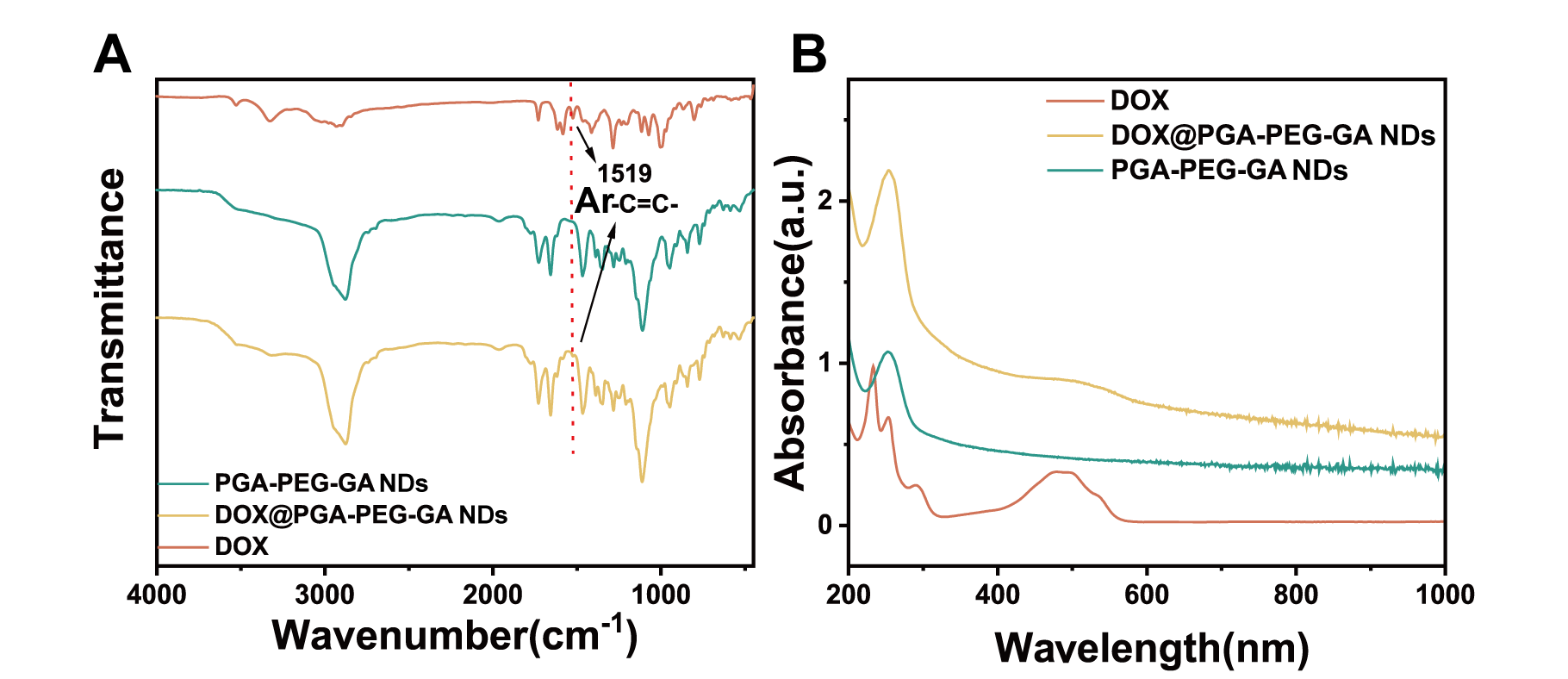


**Figure S5**. Characterization of DOX@PGA-PEG-GA NDs. (A) FT-IR spectra and (B) UV-Vis absorption spectra of DOX, PGA-PEG-GA NDs, and DOX@PGA-PEG-GA NDs.

**Table S3.** Determination of Encapsulation Efficiency (EE) and Drug-Loading Capacity (DLC) of DOX in nanodrugs

| NDs | EE (%) | DLC (%) |
| --- | --- | --- |
| DOX@PGA NDs | 81.20±1.10 | 7.53±0.06 |
| DOX@PGA-PEG NDs | 93.18±0.36 | 8.53±0.03 |
| DOX@PGA-PEG-GA NDs | 96.62±0.93 | 8.82±0.09 |


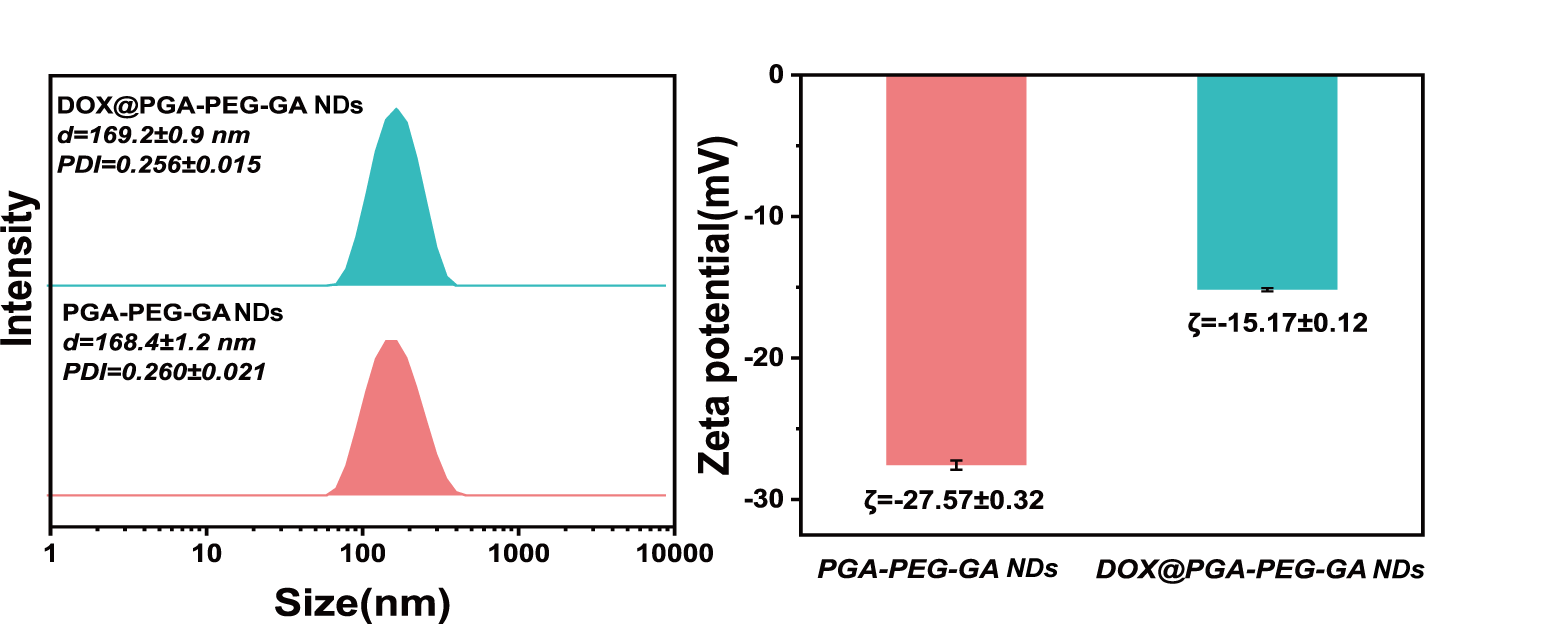


**Figure S6**. (A) Hydrodynamic diameter and (B) zeta potential of PGA-PEG-GA NDs and DOX@PGA-PEG-GA NDs.


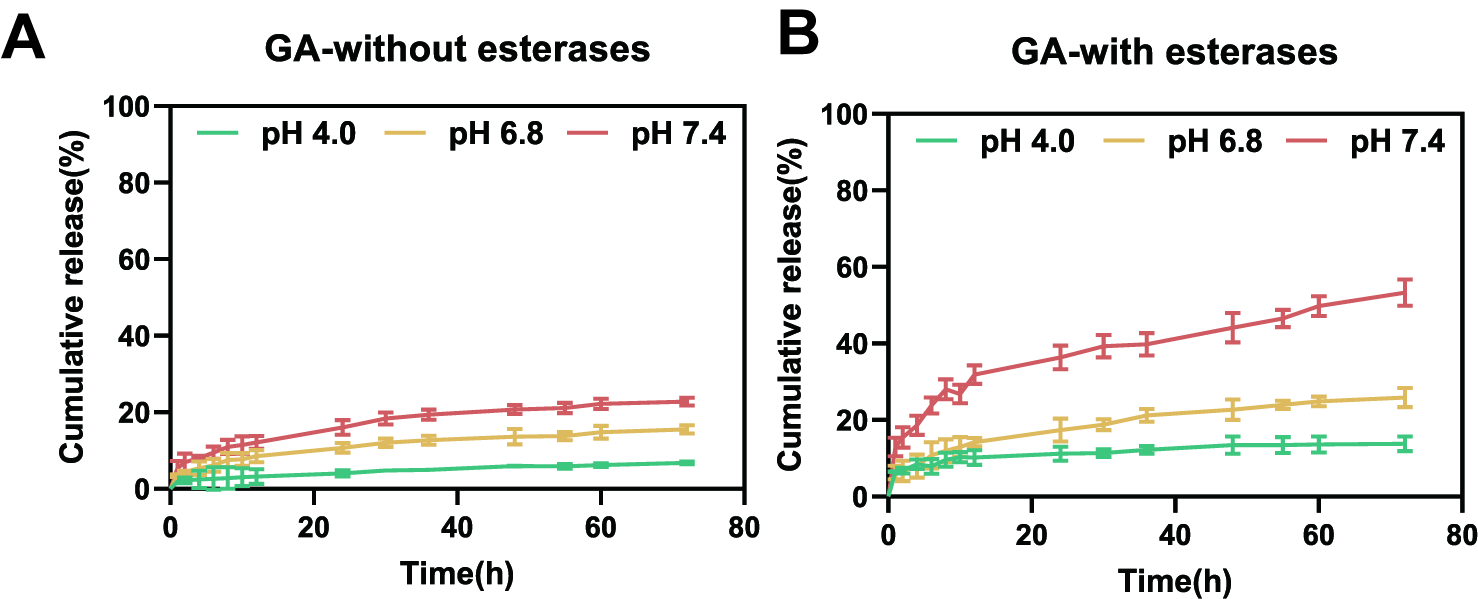


**Figure S7**. In vitro release of GA. (A-B) In vitro release of GA from DOX@PGA-PEG-GA NDs in different pH conditions A) without and B) with esterase. The data are presented as the mean ± SD.


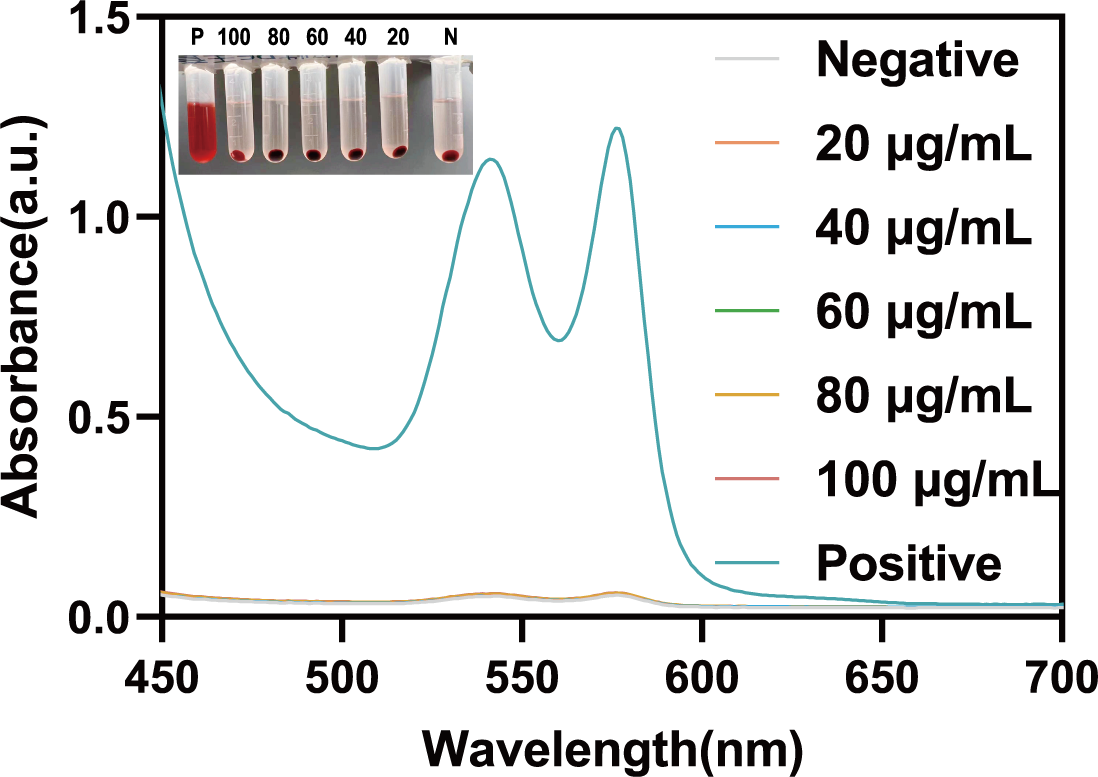


**Figure S8**. The UV-Vis absorption spectra of DOX@PGA-PEG-GA NDs with different concentrations after incubation with red blood cells.


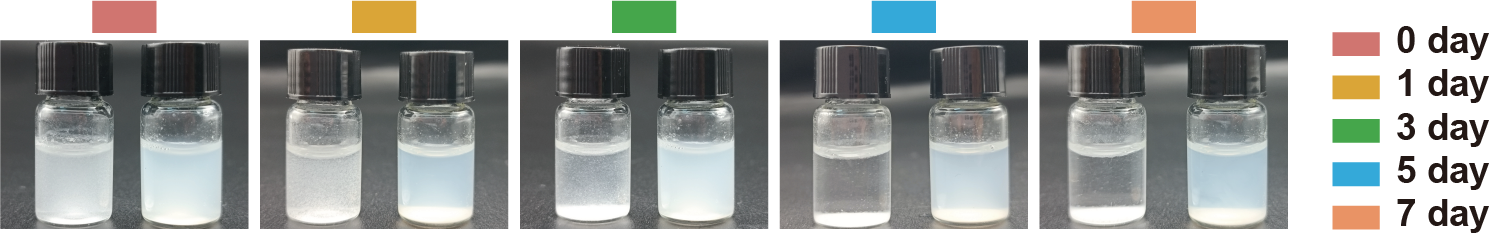


**Figure S9**. Stability studies of PGA NDs (left) and PGA-PEG-GA NDs (right)


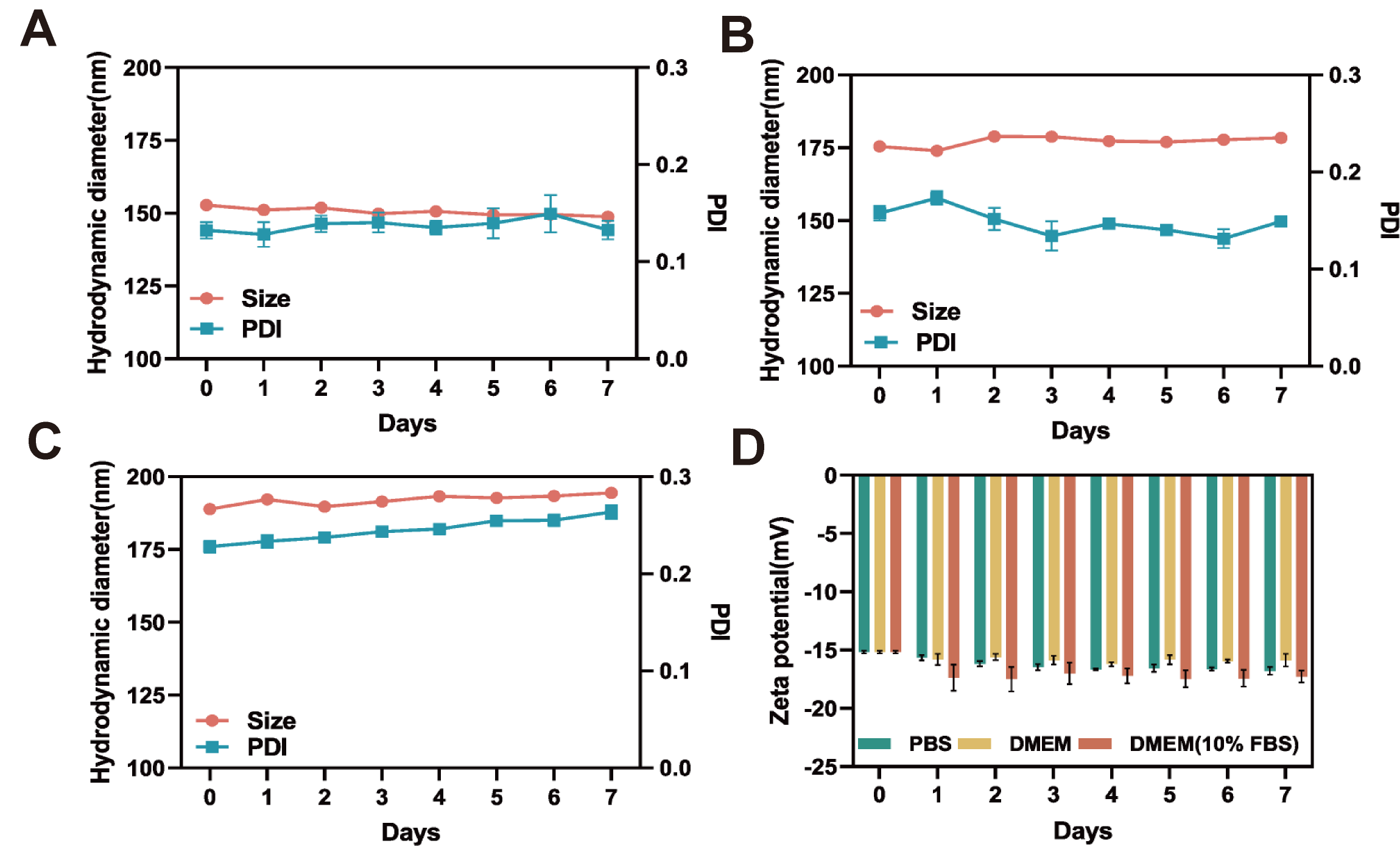


**Figure S10**. Hydrodynamic diameters and PDI of DOX@PGA-PEG-GA NDs were measured in (A) PBS, (B) DMEM cell culture medium, and (C) DMEM (10% FBS) cell culture medium on different days (n = 3). (D) Zeta potential of DOX@PGA-PEG-GA NDs measured in PBS, DMEM cell culture medium, and DMEM (10% FBS) cell culture medium on different days (n = 3). The data are presented as the mean ± SD.


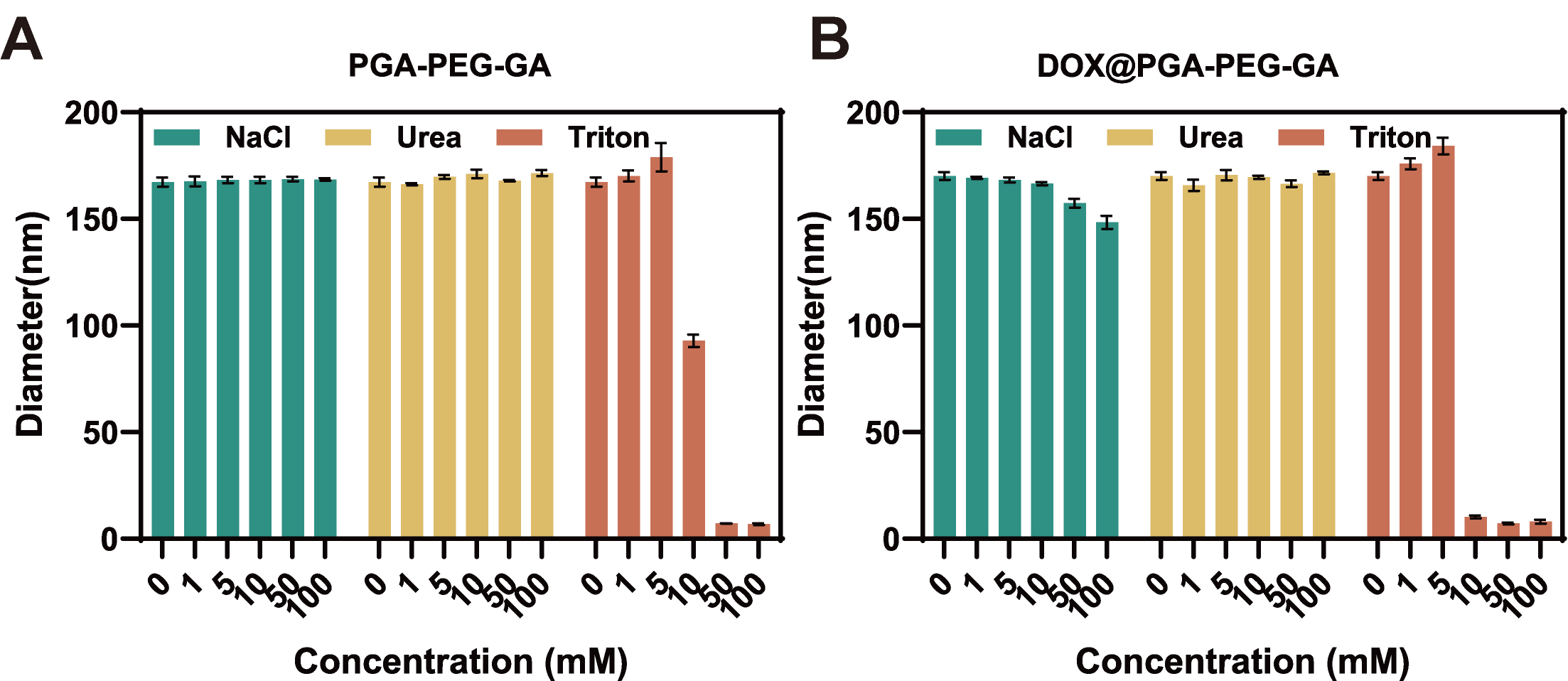


**Figure S11**. Formation mechanism of (A) PGA-PEG-GA NDs and (B) DOX@PGA-PEG-GA NDs incubated with NaCl, Urea, or Triton at different concentrations (n =3). The data are presented as the mean ± SD.


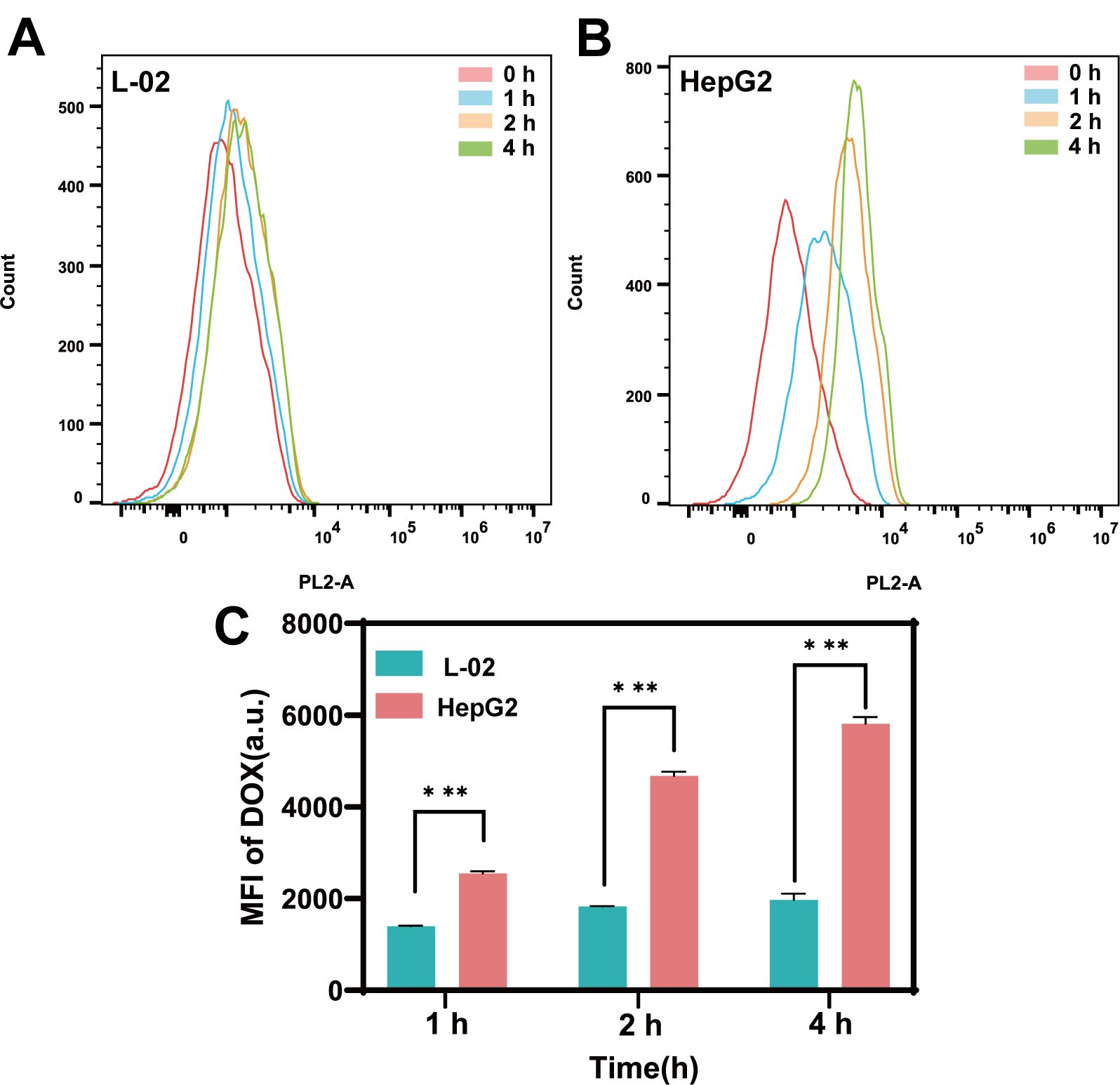


**Figure S12**. **(**A-B) Flow cytometric analysis of DOX fluorescence intensity in A) L-02 and B) HepG2 cells treated with DOX@PGA-PEG-GA NDs at 0 h, 1 h, 2 h, and 4 h. (C) Quantitative analysis of the mean fluorescence intensity (MFI) of DOX in L-02 and HepG2 cells at different time points. Data are presented as mean ± SD (n=3), with statistical significance indicated as *p < 0.05, **p < 0.01, *** p < 0.001.


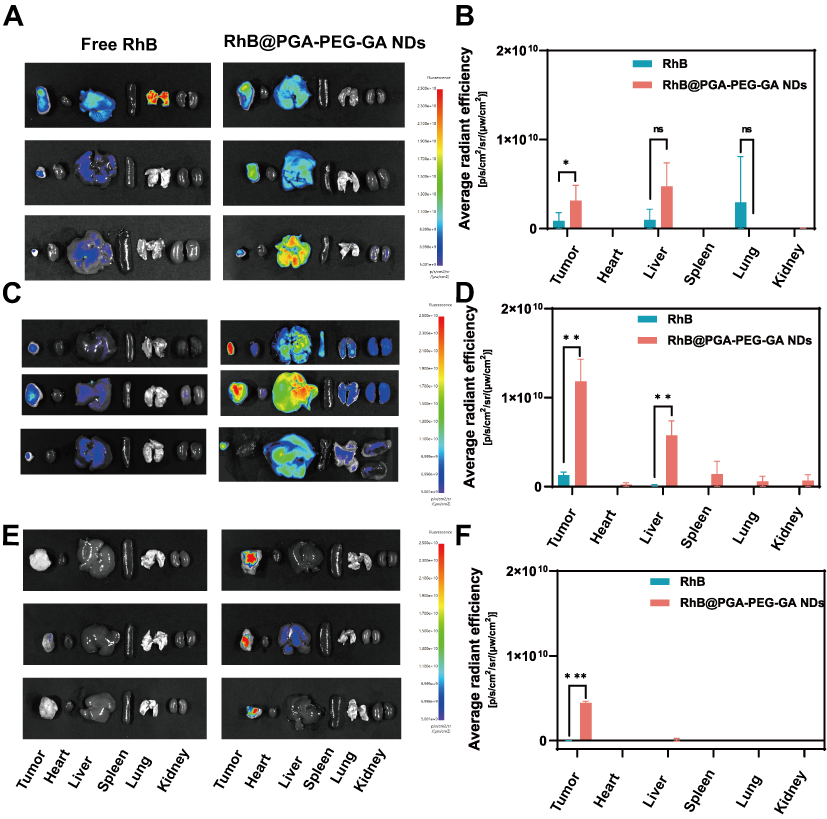


**Figure S13**. *Ex vivo* fluorescence imaging of tumor and major organs (heart, liver, spleen, lung, kidney) from mice treated with free RhB and RhB@PGA-PEG-GA NDs at 12 h (A-B), 24 h (C-D), and 48 h (E-F). The average radiant efficiency of RhB in each organ is quantified and presented as mean ± SD (n=3), with statistical significance indicated as *p < 0.05, **p < 0.01, *** p < 0.001.


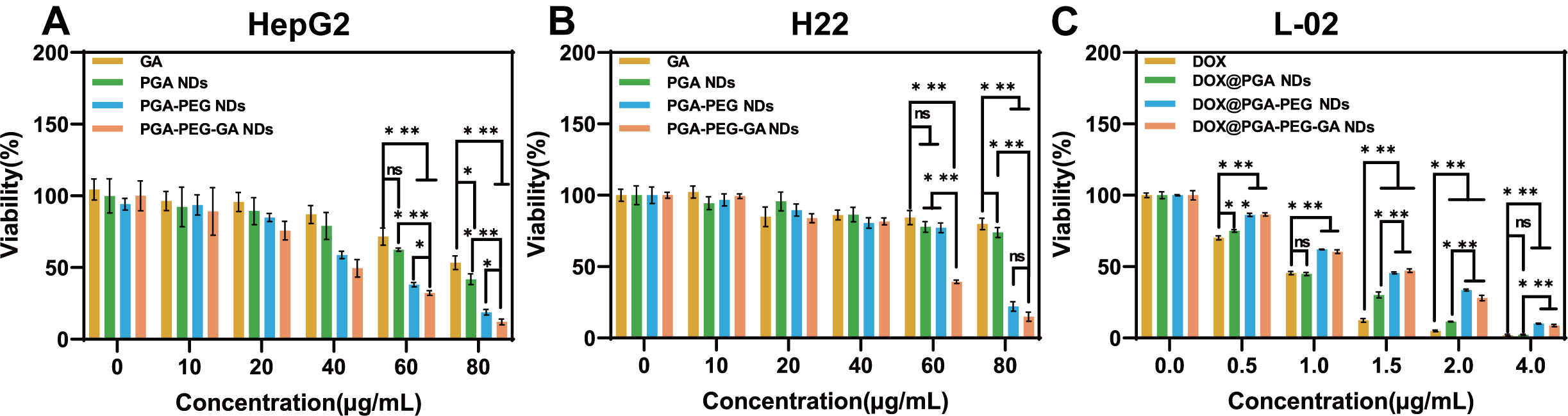


**Figure S14**. (A-B) Cell viability of A) HepG2 and B) H22 cells treated with blank nanodrugs (GA, PGA NDs, PGA-PEG NDs, PGA-PEG-GA NDs) concentrations of 0, 10,20, 40, 60, and 80 μg/mL. (C) Cell viability of L-02 cells treated with different formulations (DOX, DOX@PGA NDs, DOX@PGA-PEG NDs, DOX@PGA-PEG-GA NDs) at concentrations of 0, 0.5, 1, 2, and 4 μg/mL. Data are presented as mean ± SD (n=3), with statistical significance indicated as *p < 0.05, **p < 0.01, *** p < 0.001.

**Table S4**: IC50 Values of Different Drug Treatments on HepG2, H22, and L-02 Cell Lines

| Drug Treatment | HepG2 (μg/mL) | H22 (μg/mL) | L-02 (μg/mL) |
| --- | --- | --- | --- |
| DOX | 1.5703±0.1196 | 1.1740±0.0985 | 0.7924±0.0820 |
| DOX@PGA NDs | 0.7827±0.0703 | 0.9366±0.1133 | 0.8936±0.0533 |
| DOX@PGA-PEG NDs | 0.6809±0.0472 | 0.8209±0.1121 | 1.3427±0.0275 |
| DOX@PGA-PEG-GA NDs | 0.6091±0.0835 | 0.3760±0.0705 | 1.2997±0.0540 |


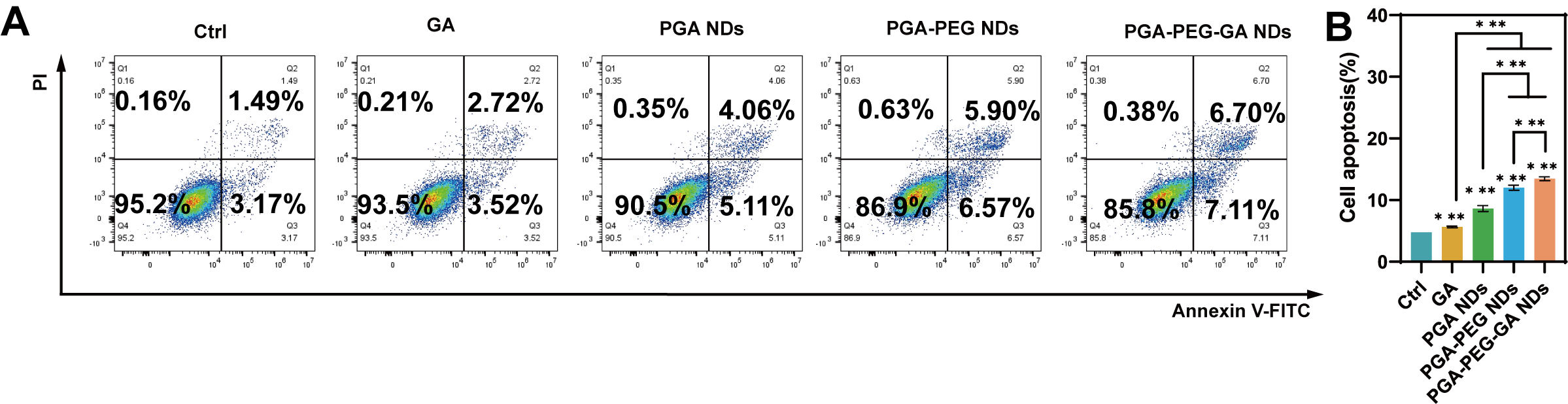


**Figure S15**. (A) Flow cytometric analysis of apoptosis in HepG2 cells treated with different blank nanodrugs. The percentages of cells in each quadrant (Q1–Q4) are indicated, representing different apoptotic stages. (B) Quantitative analysis of cell apoptosis rates. Data are presented as mean ± SD (n=3), with statistical significance indicated as *p < 0.05, **p < 0.01, *** p < 0.001.


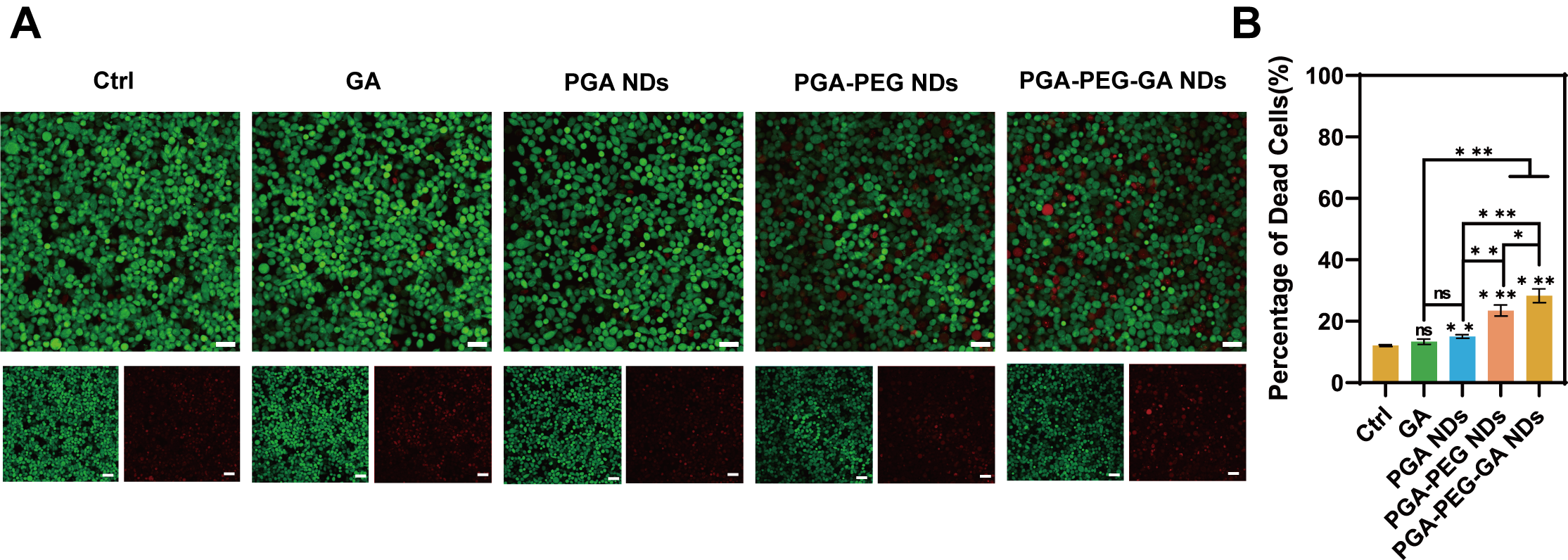


**Figure S16**. (A) Confocal microscopy images of dead cells (red fluorescence) and live cells (green fluorescence) in HepG2 cells treated with different blank nanodrugs. Scale bars: 100 µm. (B) Quantitative analysis of dead cell percentages. Data are presented as mean ± SD (n=3), with statistical significance indicated as *p < 0.05, **p < 0.01, *** p < 0.001.


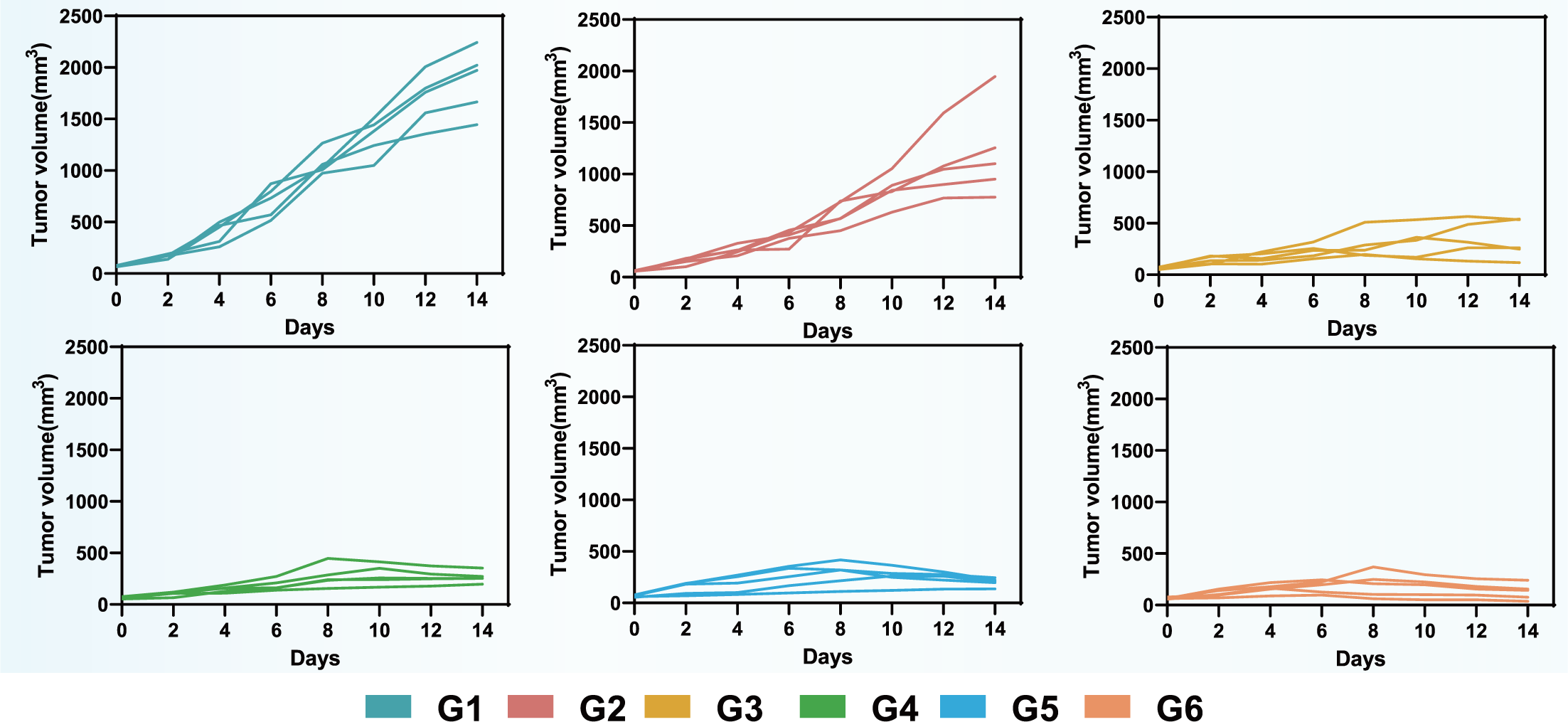


**Figure S17**. Tumor volume growth curves of G1-G6 groups. (G1: Saline, G2: PGA-PEG-GA NDs, G3: Free DOX, G4: DOX@PGA NDs, G5: DOX@PGA-PEG NDs, G6: DOX@PGA-PEG-GA NDs).


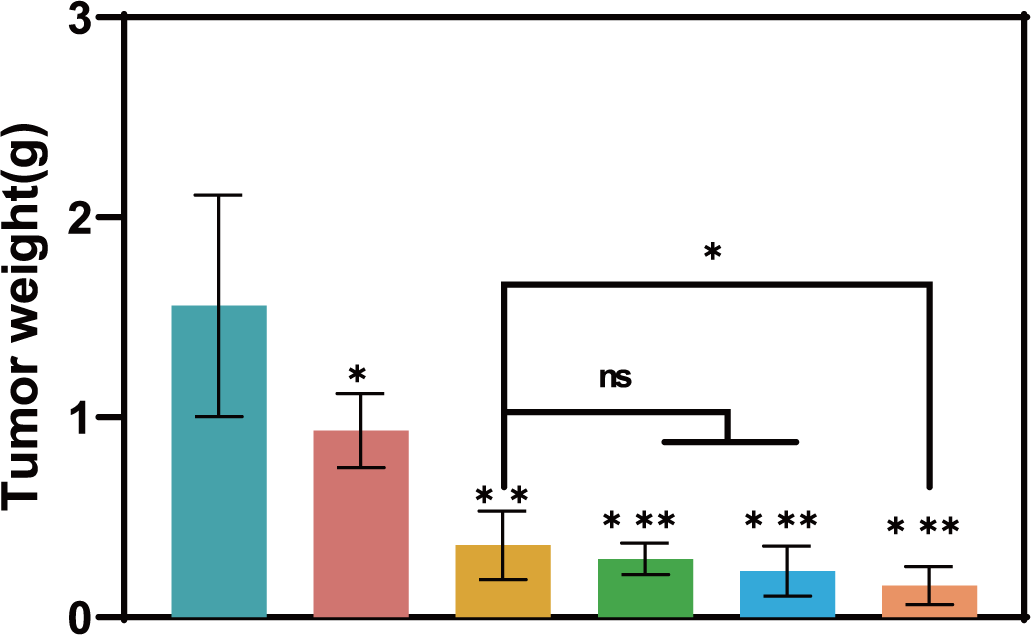


**Figure S18**. Quantitative weight assessment of solid tumors in groups G1-G6.


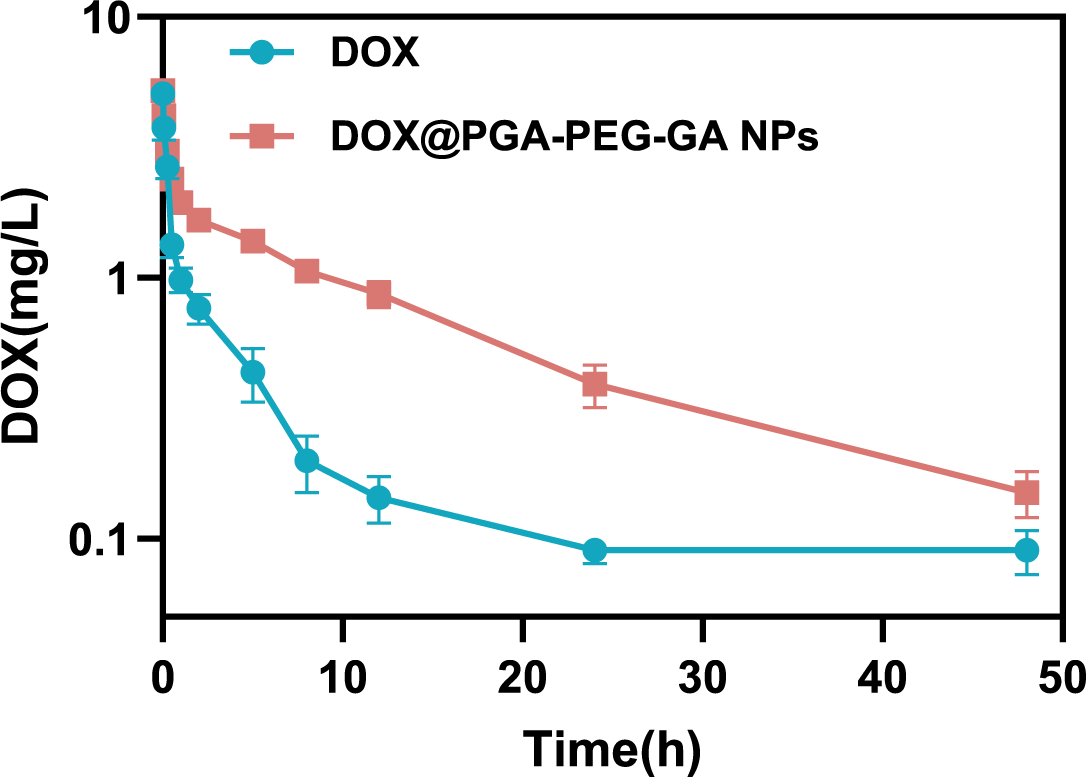


**Figure S19**. Plasma concentration of DOX after administration of free DOX and DOX@PGA-PEG-GA NDs.

**Table S5**. Pharmacokinetic results for free DOX and DOX@PGA-PEG-GA NDs

| Parameter | Free DOX | DOX@PGA-PEG-GA NDs |
| --- | --- | --- |
| Dosage [mg kg^−1^] | 5 | 5(eq.DOX) |
| t_1/2_ (h)^(a)^ | 3.50±0.39 | 10.20±0.50^(b)^ |
| CL (L·h^-1^)^(a)^ | 0.071±0.010 | 0.021±0.010^(b)^ |
| AUC(mg·h·L^-1^)^(a)^ | 2.13±0.30 | 5.84±0.64^(b)^ |
| MRT(h)^(a)^ | 0.43±0.06 | 1.30±0.23^(b)^ |

(a) Abbreviations—t_1/2_: Half-life time; CL: Clearance rate; AUC: Area under the curve; MRT: Mean residence time.

(b) p < 0.001 compared to the free DOX group.


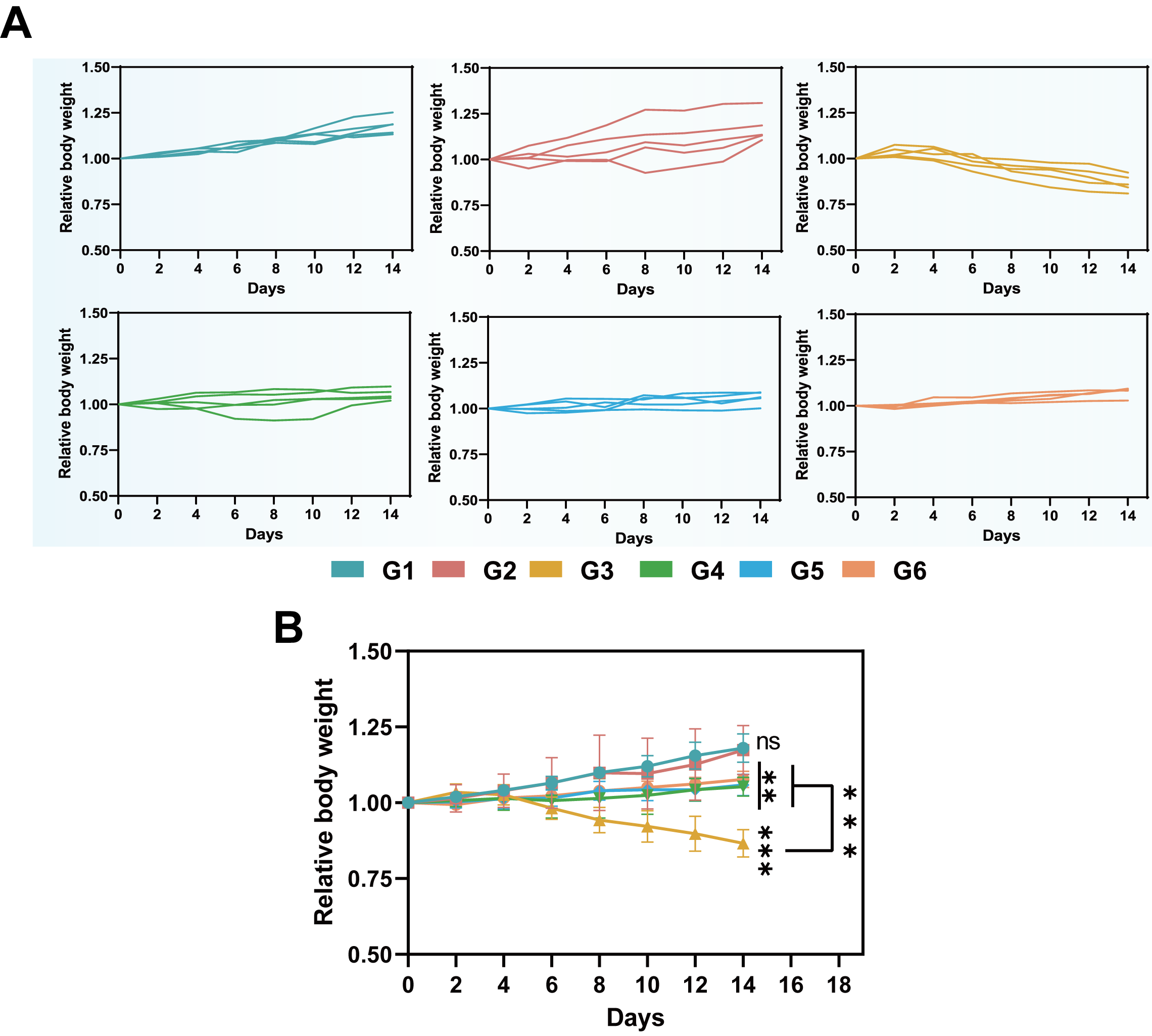


**Figure S20**. (A) Relative body weight changes in different treatment groups (G1: Saline, G2: PGA-PEG-GA NDs, G3: Free DOX, G4: DOX@PGA NDs, G5: DOX@PGA-PEG NDs, G6: DOX@PGA-PEG-GA NDs). Each line represents the relative body weight change of an individual mouse over 14 days. (B) Relative body weight changes over time in different treatment groups. Data are presented as mean ± SD (n=5), with statistical significance indicated as *p < 0.05, **p < 0.01, ***p < 0.001.


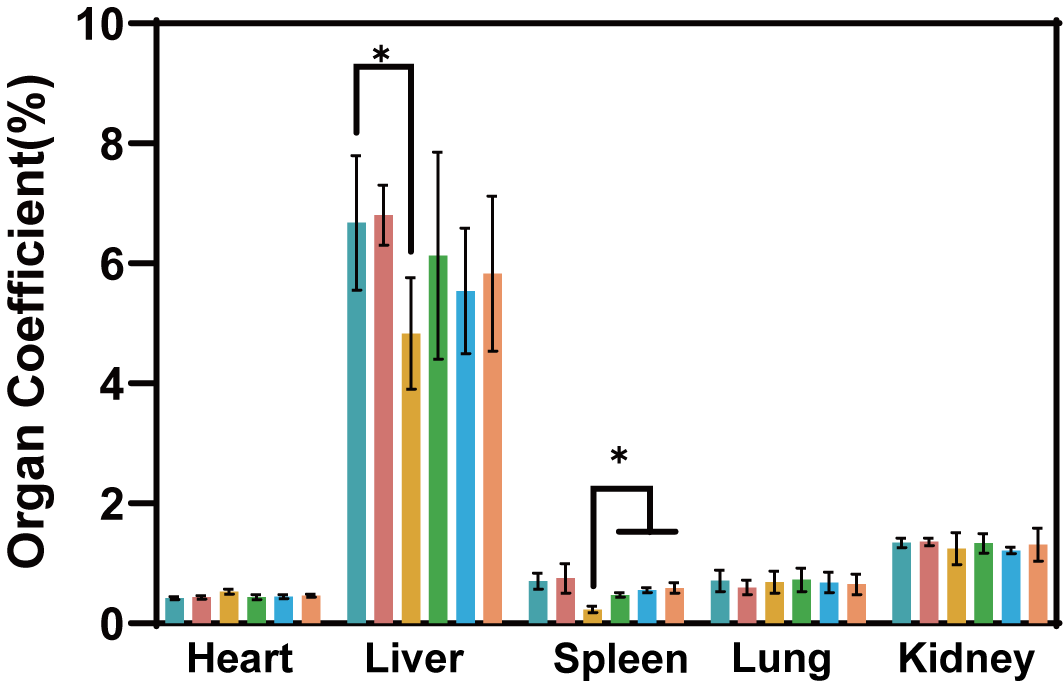


**Figure S21**. Organ coefficient in groups of mice after different drug treatments.


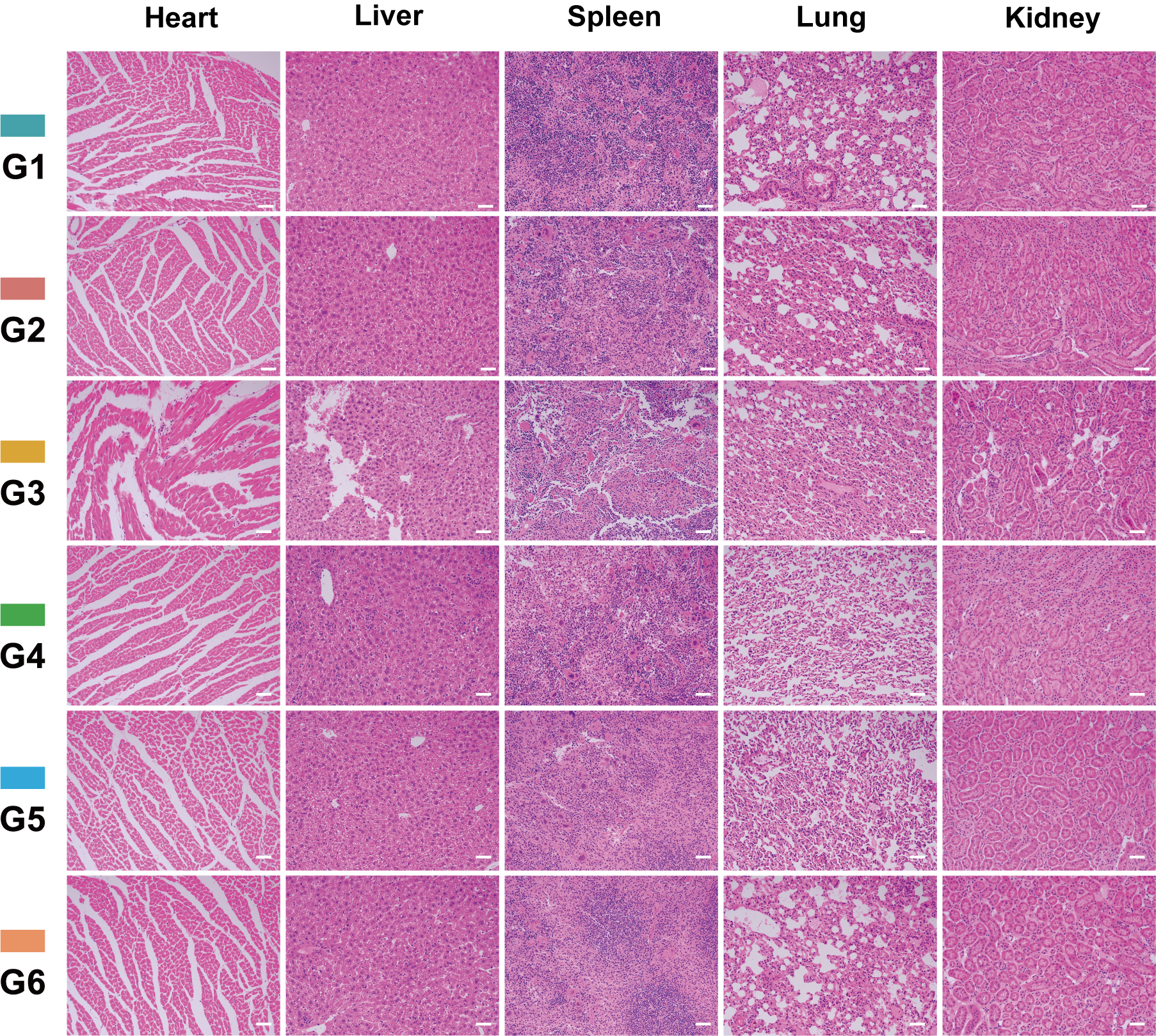


**Figure S22**. HE staining of major organs. Scale bars: 100 μm (G1: Saline, G2: PGA-PEG-GA NDs, G3: Free DOX, G4: DOX@PGA NDs, G5: DOX@PGA-PEG NDs, G6: DOX@PGA-PEG-GA NDs).


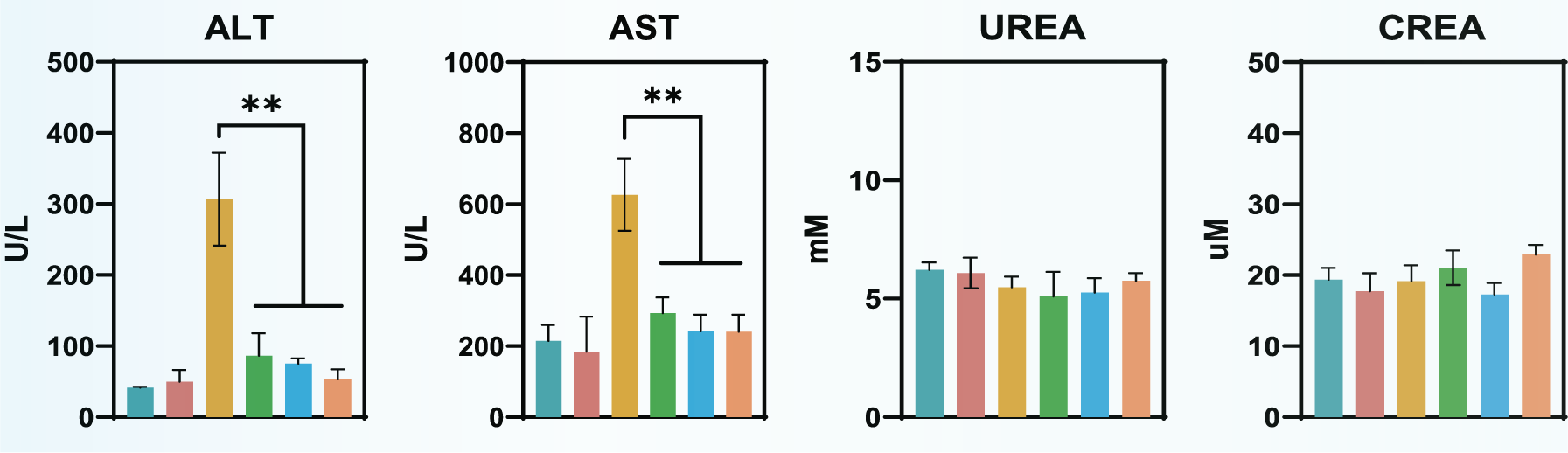


**Figure S23**. Biochemical indices after different drug treatments.


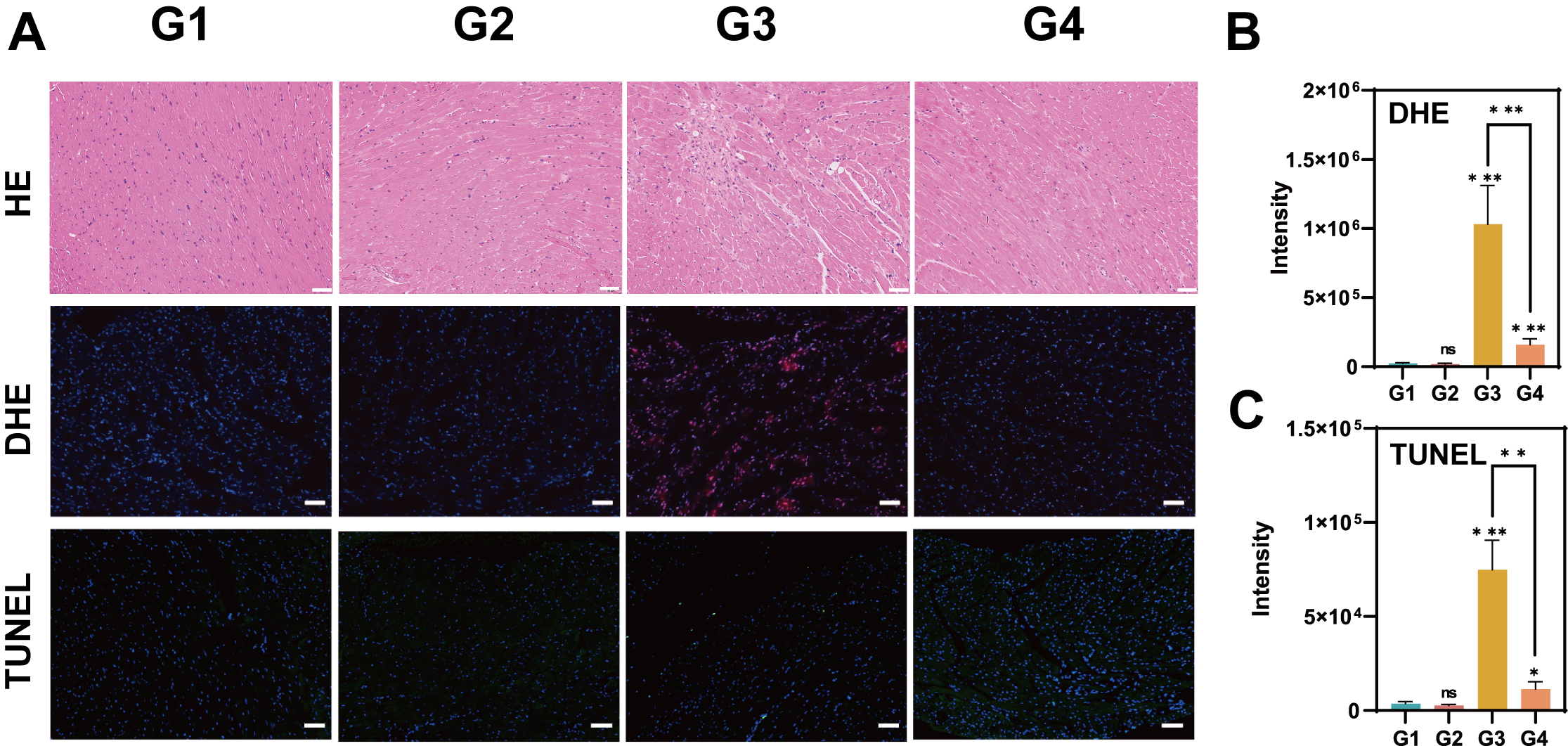


**Figure S24**. (A) Histological analysis of heart tissues from different groups (G1: Saline, G2: PGA-PEG-GA NDs, G3: Free DOX, G4: DOX@PGA-PEG-GA NDs). HE staining, DHE staining, and TUNEL staining are shown. Scale bars: 100 μm. (B) Quantitative analysis of DHE fluorescence intensity in heart tissues from different groups. (C) Quantitative analysis of TUNEL fluorescence intensity in heart tissues from different groups. Data are presented as mean ± SD (n=3), with statistical significance indicated as *p < 0.05, **p < 0.01, *** p < 0.001.
